# Multifunctional composite magnetic particles combined with static magnetic field for treating infectious bone defects via *SLC2A1*-mediated HIF-1 and PI3K/AKT pathways

**DOI:** 10.1186/s11671-026-04923-w

**Published:** 2026-10-31

**Authors:** Mingming Huang, Yuqi Li, Ludan Qin, Xin Gao, Yiyang Tang, Tian-Yun Huang, Jianmin Han, Menglong Hu

**Affiliations:** 1 Department of Prosthodontics, National Center for Stomatology & National Clinical Research Center for Oral Diseases & National Engineering Research Center of Oral Biomaterials and Digital Medical Devices& Beijing Key Laboratory of Digital Stomatology & NHC Key Laboratory of Digital Stomatology & NMPA Key Laboratory for Dental Materials, Peking University School and Hospital of Stomatology, Beijing, PR China; 2 School and Hospital of Stomatology & Tianjin Key Laboratory of Oral Soft and Hard Tissues Restoration and Regeneration, Tianjin Medical University, Tianjin, PR China; 3 Department of Prosthodontics, School and Hospital of Stomatology, Cheeloo College of Medicine, Shandong Key Laboratory of Oral Tissue Regeneration & Shandong Engineering Laboratory for Dental Materials and Oral Tissue Regeneration & Shandong Provincial Clinical Research Center for Oral Diseases, Shandong University, Jinan, PR China; 4 Laboratory for Micromachine Intelligence, School of Advanced Manufacturing and Robotics, PKU Research Center for Robotics, Peking University, Beijing, PR China; 5 National Key Laboratory of Advanced Micro and Nano Manufacture Technology, Beijing, PR China; 6 Department of Prosthodontics, Affiliated Hospital of Medical School, Institute of Stomatology, Nanjing Stomatological Hospital, Nanjing University, Nanjing, 210008 PR China

**Keywords:** Magnetic particles, Static magnetic field, Antibacterial effect, Bone regeneration, Infectious bone defect

## Abstract

Infectious bone defects are a major challenge in orthopedics and stomatology, requiring strategies that eliminate bacteria and promote osteogenesis. Here, we developed a multifunctional composite magnetic particle system (F-Fe_3_O_4_) and demonstrate its synergistic combination with a static magnetic field (SMF) for treating infectious bone defects. In vitro, F-Fe_3_O_4_ demonstrated good biocompatibility with human bone marrow-derived mesenchymal stem cells (hBMSCs) and facilitated their proliferation, migration, and osteogenic differentiation, while inhibiting the growth of *Escherichia coli*,* Staphylococcus aureus*,* and Enterococcus faecalis*. With the application with SMF, the aforementioned effects are further enhanced. The in vivo studies in the rat model further revealed that F-Fe_3_O_4_ combined with SMF can reduce early infection, alleviate inflammation, and accelerate new bone formation. Mechanistically, RNA sequencing and verification experiments revealed that the combination therapy upregulated *SLC2A1*-mediated glucose uptake and activated HIF-1 and PI3K/AKT signaling pathways, thereby enhancing the osteogenic differentiation of hBMSCs. In conclusion, this study developed an efficient treatment approach for infectious bone defects by integrating F-Fe_3_O_4_ and SMF.

**Supplementary Information:**

The online version contains supplementary material available at https://doi.org/10.1186/s11671-026-04923-w.

## Introduction

Infectious bone defects, frequently resulting from osteomyelitis or trauma, pose a major challenge for clinicians in orthopedics and stomatology [1, 2]. Current therapies emphasize infection control and osteogenesis, typically involving antibiotic administration, surgical intervention, and bone grafts or implanted scaffolds [3, 4]. However, these approaches present disadvantages, such as prolonged surgeries, limited donors, poor integration, and immune rejection [5, 6]. In the context of widespread antibiotic overuse and escalating drug resistance, these challenges further complicate infection control and bone repair while increasing medical burden [7].

Advances in nanotechnology provide new opportunities for managing infectious bone defects through nanomaterials’ high specific surface area and tunable design [8, 9]. Various nanoparticles, including metals and metal oxides, e.g., gold, silver, copper oxide (CuO), and zinc oxide, as well as organic agents such as chitosan (CS), antibacterial peptides, and lysozyme, have been developed as antibiotic alternatives [10–12]. Although these materials exhibit potent antibacterial activity, they also have limitations, including potential biotoxicity and unstable release of active components, which restrict clinical translation [13]. Furthermore, conventional strategies often fail to maintain effective concentrations at poorly vascularized infection sites, where prolonged inflammation worsens the local microenvironment through reduced pH, hypoxia, increased oxidative stress, and negative potential, disrupting the balance between bone formation and resorption while hindering healing [14–17]. Moreover, the strong antibacterial actions required for infection control (e.g., high local doses and aggressive bactericidal effects) may conflict with the mild, supportive microenvironment needed for tissue regeneration [18]. Therefore, developing an integrated therapeutic strategy that targets infection sites, sustains local concentration, and balances antibacterial and osteogenic functions is essential.

Given their unique magnetic properties, favorable biosafety, and easy surface modification, biomedical magnetic nanomaterials provide an ideal platform for constructing multifunctional systems and have been widely studied in drug delivery, hyperthermia, and magnetic particle imaging [19, 20]. Magnetic materials, such as iron oxide Fe_3_O_4_, can be directed and concentrated at target regions using an external magnetic field [21]. Compared with passive diffusion, such externally regulated systems are better suited to enhancing local dosing in deep, poorly vascularized, and anatomically complex infection sites [22–24]. Furthermore, their surfaces facilitate versatile functionalization, thereby enabling integration of features, including antibacterial and regenerative modules, through multilayer designs to achieve synergistic effects within a single platform [25, 26].

Notably, an external magnetic field not only enriches and localizes magnetic particles at the target site but also modulates osteogenic behavior through its stimulation by affecting cell adhesion, cytoskeletal remodeling, and signaling pathways [27–29]. Alternating magnetic fields (AMFs) are commonly used in magnetic hyperthermia; however, coupled electrical and thermal effects complicate interpretation of biological responses and may introduce cytotoxicity and uncertainty [7, 30]. In contrast, static magnetic fields (SMFs) are relatively mild, noninvasive, biocompatible, and require simpler equipment, making them more appropriate for long-term or repeated applications. The effects of SMF, as a continuously applied physical stimulus, are not governed by a single pathway and can synergize with the material systems. They can influence various stem cells and osteogenesis-related cells and may promote osteogenesis via the BMP/Smad, Wnt/β-catenin, Piezo1, or MAPK pathways [27, 31], which also indicates that the mechanisms proposed in different studies are not entirely consistent and may depend on magnetic-field parameters, exposure conditions, and the intrinsic physicochemical properties of the materials. Santos et al. [32] indicated that SMF stimulation of SMF can drive osteogenesis in non-bone environments, which demonstrated the possibility of remote-controlled modulation of stem cell fate in vivo without biochemical supplementation for the first time. Moreover, it has been reported that SMF could improve bactericidal efficacy by promoting particle aggregation, strengthening interfacial contact, and increasing reactive oxygen species (ROS) generation [30, 33, 34]. However, evidence supporting SMF-associated enhancement of antibacterial activity remains relatively limited and inconsistent. SMF combined with magnetic biomaterials has been reported to enhance antibacterial activity against both Gram-negative and Gram-positive bacteria, although this enhancement effect was not accompanied by an increase in ROS, suggesting that other mechanisms may be involved besides oxidative stress [35]. Chen et al. [36] developed a ZnFe_2_O_4_-based magnetic scaffold that, under 30 mT SMF, promoted cell proliferation, differentiation and mineralization while providing ROS-scavenging and antibacterial effects through its functional components and ion release. However, these findings were limited to in vitro evaluation and require validation in infected bone-defect models in vivo. Present evidence supporting antibacterial and osteogenic enhancement by SMFs combined with magnetic particles in infectious bone defects remains limited, with several studies addressing only single endpoints or being limited in vitro [24, 32, 33, 36, 37]. A coherent chain of evidence and clear mechanistic links from material design to bacterial inhibition and cellular response is still lacking.

**Scheme 1:**
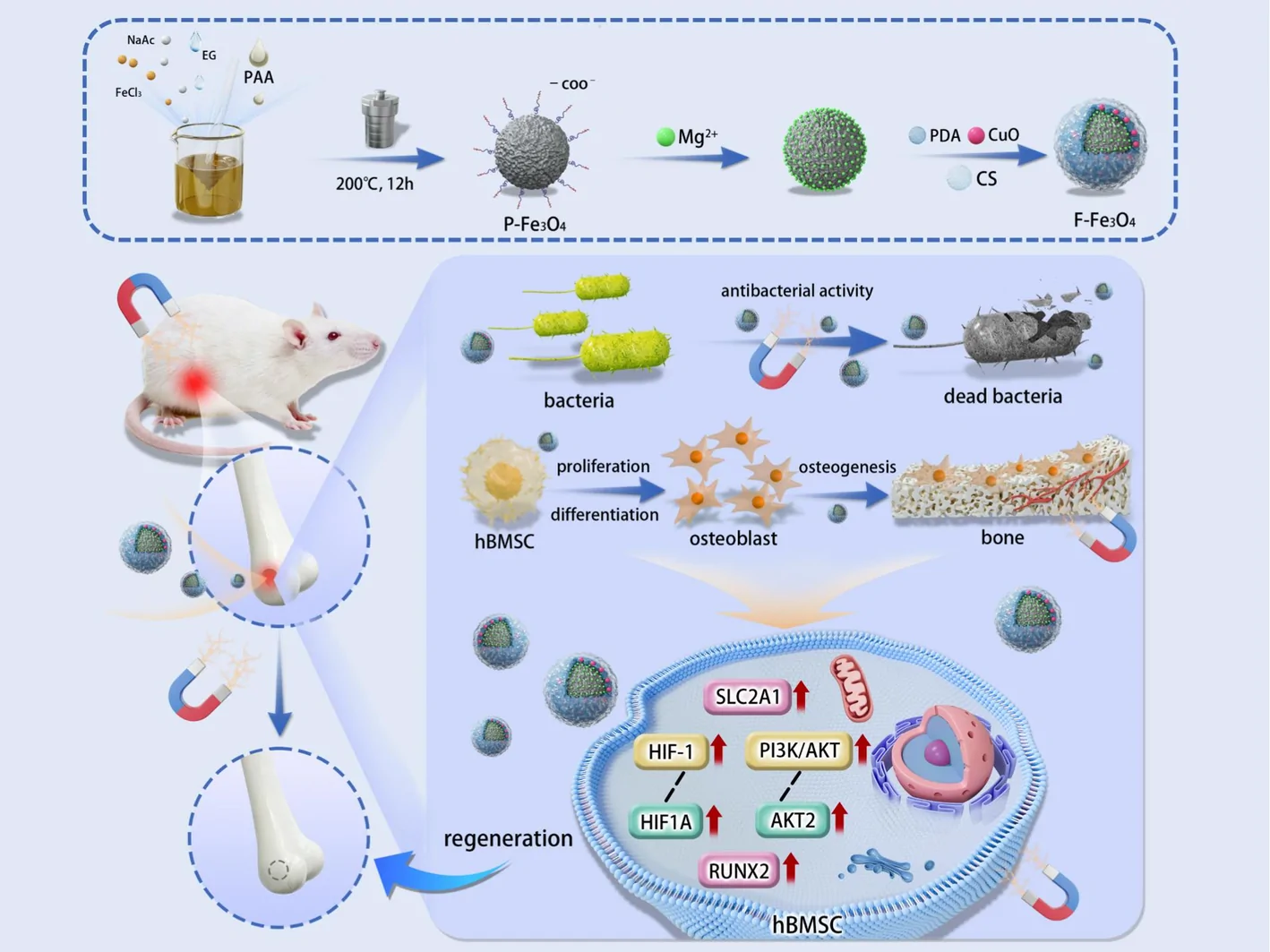
Schematic illustration of the preparation of the multifunctional composite magnetic particle system F-Fe_3_O_4_ and its role in treating infectious bone defects under the static magnetic field

Accordingly, we developed a multilayered, multifunctional magnetic particle system (Scheme 1). Poly(acrylic acid) (PAA)-modified Fe_3_O_4_ (P-Fe_3_O_4_) served as the magnetic core and provided surface functional groups for subsequent hierarchical assembly [38, 39]. Magnesium ions (Mg^2+^), polydopamine (PDA), CuO, and CS were sequentially incorporated to construct the final composite, P-Fe_3_O_4_-Mg^2+^@PDA-CuO@CS (abbreviated as F-Fe_3_O_4_), thereby integrating magnetic responsiveness with osteogenesis-supportive, antibacterial, and biocompatible functionalities within a single platform [18, 40–42]. We further combined F-Fe_3_O_4_ with SMF as an external physical stimulus and evaluated its antibacterial and osteogenic performance both in vitro and invivo*.* For the in vivo rat model, F-Fe_3_O_4_ was loaded into the injectable methacrylate gelatin (GelMA) hydrogel to facilitate direct local delivery and retention of the magnetic composite particles at the defect site, taking advantage of the good biocompatibility and in situ gelation properties of GelMA. In addition, the molecular mechanisms underlying the enhanced osteogenic response to F-Fe_3_O_4_+SMF were investigated. Overall, this study provides a multifunctional magnetic material strategy integrating antibacterial and osteogenic functions with external magnetic-field stimulation and experimental evidence for its potential application in treating infectious bone defects.

## Materials and methods

### Synthesis of the Fe_3_O_4_ and P-Fe_3_O_4_ particles

A one-pot solvothermal method was used to synthesize Fe_3_O_4_ and P-Fe_3_O_4_ particles, based on previous studies [43, 44]. Iron(III) chloride hexahydrate (FeCl_3_•6H_2_O, 4.05 g), sodium acetate anhydrous (NaAc, 10.8 g), and ethylene glycol (EG, 120 mL) were mixed and stirred overnight. The mixture was then transferred into a Teflon-lined stainless-steel autoclave and reacted at 200℃ for 12 h. The precipitate was collected using the neodymium-iron-boron (NdFeB) magnet, washed, and vacuum-dried at room temperature (RT) to obtain Fe_3_O_4_. Similarly, FeCl_3_•6H_2_O, NaAc, and EG were mixed, added with PAA (0.125 g), and stirred overnight. P-Fe_3_O_4_ was obtained after the same treatment. Aladdin (China) supplied FeCl_3_•6H_2_O, NaAc, and EG. Sigma-Aldrich (USA) provided PAA.

### Synthesis of the F-Fe_3_O_4_ particles

P-Fe_3_O_4_ (50 mg) was dispersed in a 0.2-mol/L aqueous magnesium chloride (MgCl_2_) solution, and sonicated for 0.5 h, then mechanically stirred for 4 h [39, 45]. The product was then dispersed in 10 mM Tris-HCl buffer, sonicated for 0.5 h, followed by the addition of dopamine, and stirred for 1 h [46]. Subsequently, CuO nanoparticles (≤ 50 nm; Sigma-Aldrich) were added, followed by ultrasonication for 0.5 h and stirring for 2 h [42], and P-Fe_3_O_4_-Mg^2+^@PDA-CuO was obtained. CS was dissolved in a prepared 1% acetic acid solution and stirred at 50℃ for 1 h until the solution became clear and transparent. The pH was adjusted with NaOH, and the solution was filtered. P-Fe_3_O_4_-Mg^2+^@PDA-CuO was added to the filtrate and dispersed uniformly. The 5% (v/v) glutaraldehyde was added, and the reaction was conducted for 2 h [47]. The product was washed alternately with deionized water and ethanol to remove residual glutaraldehyde before subsequent experiments and dried under vacuum at RT to obtain the final functionalized magnetic particles, designated as F-Fe_3_O_4_. MgCl_2_, dopamine, CS, and glutaraldehyde were procured from Aladdin.

### Characterization

Scanning electron microscope (SEM; ZEISS, Germany) and transmission electron microscope (TEM; FEI, USA) were used to analyze the microstructures of Fe_3_O_4_, P-Fe_3_O_4_, and F-Fe_3_O_4_. Energy-dispersive X-ray spectrometer (EDS; Oxford Instruments, UK) was used to analyze the elemental composition and distribution of F-Fe_3_O_4_. F-Fe_3_O_4_ was supported on molybdenum (Mo) grids for TEM observation. The specific surface area, pore-size distribution, and porosity of Fe_3_O_4_ and P-Fe_3_O_4_ were measured using an automated surface area and porosity analyzer (ASAP 2460, Micromeritics, USA) based on the Brunauer–Emmett–Teller (BET) method. A dynamic light scattering (DLS) and zeta potential analyzer (Malvern, UK) were used to measure the hydrodynamic size distribution and zeta potential of Fe_3_O_4_, P-Fe_3_O_4_, and F-Fe_3_O_4_. The crystal structure and phase composition of the particles were characterized through X-ray diffractometer (XRD; D8 Advance, Bruker, Germany). A vibrating sample magnetometer (VSM; 8604, Lake Shore, USA) was used to measure the magnetic properties of P-Fe_3_O_4_ and F-Fe_3_O_4_. The thermal stability and mass-loss behavior of P-Fe_3_O_4_ and F-Fe_3_O_4_ were evaluated through thermogravimetric analyzer (TGA; STA 8000, PerkinElmer, USA). Fourier transform infrared spectrometer (FT-IR; Thermo Fisher Scientific, USA) was used to analyze functional groups. Inductively coupled plasma mass spectrometer (ICP-MS) and ICP optical emission spectrometer (both Agilent, USA) were used to quantify the ion-release behavior of F-Fe_3_O_4_ under physiological conditions (Phosphate Buffer Saline, 37 °C). X-ray photoelectron spectrometer (XPS; Thermo Scientific) was used to analyze the surface elemental composition of the particles.

### Cell culture

Human bone marrow-derived mesenchymal stem cells (hBMSCs) (ScienCell Company, USA) were cultured at 37℃ in a 5% CO_2_ humidified atmosphere. The proliferation medium (PM) contained 10% fetal bovine serum (FBS) and 1% penicillin–streptomycin mixture in α-minimum essential medium (α-MEM). The osteogenic medium (OM) contained 10% FBS, 1% penicillin–streptomycin mixture, 10 nM dexamethasone, 200 µM L-ascorbic acid, and 10 mM β-glycerophosphate in α-MEM. α-MEM, FBS, and penicillin–streptomycin mixture were procured from Gibco (USA). Sigma-Aldrich provided dexamethasone, L-ascorbic acid, and β-glycerophosphate.

### Cell biocompatibility and proliferation assay

Moreover, hBMSCs were seeded in well plates and incubated overnight in PM, and the medium was replaced based on different treatments on the following day. A working concentration of 60 µg/mL F-Fe_3_O_4_ was selected following preliminary screening of 20–100 µg/mL based on qualitative microscopic assessment of cell morphology, as 60 µg/mL was the highest concentration without obvious adverse morphological effects and ensuring biological activity. Four groups were settled: the PM group received particle-free PM; the P-Fe_3_O_4_ group received PM containing 60 µg/mL P-Fe_3_O_4_; and the F-Fe_3_O_4_ group received PM containing 60 µg/mL F-Fe_3_O_4_. Cells were cultured in PM containing 60 µg/mL F-Fe_3_O_4_ with external SMF to evaluate magnetic effects in the F-Fe_3_O_4_+SMF group. NdFeB permanent magnets (10 mm in diameter and 20 mm in height) were placed at the fixed position beneath the culture dish. The magnetic field strength at different points on the cell level of the culture dish was measured using a Gauss meter (DELIXI, China) and was approximately 100 mT, with continuous exposure throughout the experiment [48–50].

Cell morphology and viability were assessed after 24 h using the Live/Dead staining kit (KeyGen Biotech, China) and observed under a fluorescence microscope (BX53, Olympus, Japan). Live cells emitted green fluorescence, whereas dead cells emitted red, under fluorescence excitation.

The cytoskeleton was observed 24 h post-incubation. Cells were fixed with 4% paraformaldehyde (Biosharp, China), permeabilized with 0.2% Triton X-100 (Sigma-Aldrich), and stained with phalloidin and DAPI (Thermo Fisher Scientific) to visualize F-actin and cell nuclei, respectively. A confocal laser scanning microscope (CLSM; FV300, Olympus, Japan) was used for observation.

Cell proliferation was assessed on days 0, 1, 3, 5, and 7 using 10% Cell Counting Kit-8 (CCK-8; Dojindo, Japan) solution. A microplate reader (Molecular Devices, USA) was used to measure the optical density (OD) values of the supernatant at 450 nm.

### Cell migration assay

The scratch assay was performed to evaluate the horizontal migration capacity of hBMSCs in vitro [51]. When the fusion rate of hBMSCs in 6-well plates reached 80%–90%, a 200-µL pipette tip was used to create scratches, and the corresponding culture medium of the four groups (PM, P-Fe_3_O_4_, F-Fe_3_O_4_, and F-Fe_3_O_4_+SMF) was added. Wound area images were captured at 0, 12, and 24 h using an optical microscope (Nikon, Japan). ImageJ software (National Institutes of Health, USA) was used to quantify the proportion of scratch closure.

The vertical migration ability was evaluated through the Transwell assay [15]. Furthermore, hBMSCs in serum-free medium were placed into the upper chamber of Transwell inserts (Corning, USA). The corresponding medium of the same four groups was added to the lower chamber. After 24 h, non-migrated cells on the upper surface of the membrane were removed using a cotton swab. The migrated cells on the lower surface were fixed with paraformaldehyde and stained with crystal violet staining solution (Beyotime, China). Images were captured under an optical microscope, and the migration was quantified by counting cells.

### Intracellular ferric ions (Fe^3+^) detection

Intracellular Fe^3+^ was detected using a Prussian blue (PB) staining kit (Yeasen, China) after coculturing for 24 h [52]. The staining result was observed under the optical microscope, in which hematoxylin or Fe^3+^ appeared blue.

### In vitro antibacterial experiments

*Escherichia coli* (*E. coli*, CMCC(B)44102), *Staphylococcus aureus* (*S. aureus*, CMCC(B)26003), and *Enterococcus faecalis* (*E. faecalis*, ATCC29212) were selected for assessing antibacterial performance. *E. coli* was cultured in Luria–Bertani broth (LB; Hopebio, China) at 37℃ for 12 h, whereas *S. aureus* and *E. faecalis* were cultured in Brain Heart Infusion broth (BHI; OXOID, UK). The bacterial suspensions were diluted with the corresponding medium to a final concentration of 1 × 10^6^ colony-forming units (CFU)/mL for subsequent experiments.

For each group, 100 µL of bacterial suspension was mixed with an equal volume of particle-containing suspension in the 96-well plate and cocultured for 12 h. The Con group received particle-free medium. The final nanoparticle concentration was 60 µg/mL in the P-Fe_3_O_4_, F-Fe_3_O_4_, and F-Fe_3_O_4_+SMF groups. OD values at 600 nm were measured every 0.5 h using a microplate reader to record bacterial growth.

The cocultured suspensions of each group were serially diluted and uniformly spread onto LB/BHI agar plates utilizing a spiral plating system (easySpiral, Interscience, France). Plates were photographed and colonies were counted 12 h post-incubation to assess bacterial viability.

To obtain SEM images, bacteria of each group were fixed with glutaraldehyde, dehydrated using increasing ethanol concentrations, and dried and coated with gold for morphological observation.

The SYTO 9/Propidium Iodide Live/Dead bacterial viability kit (Thermo Fisher Scientific) was utilized to visualize bacterial viability. The cocultured suspension was stained with the double stain in the dark and observed using CLSM 12 h post-incubation. ImageJ was used to quantify the fluorescence intensity of the bacteria in the fluorescence microscopy images.

### In vitro osteogenic ability

The medium was replaced to initiate osteogenic induction when hBMSCs reached 70%–80% confluence. The PM group received particle-free PM, the OM group received particle-free OM, the P-Fe_3_O_4_ group received OM containing 60 µg/mL P-Fe_3_O_4_, and the F-Fe_3_O_4_ and F-Fe_3_O_4_+SMF groups received OM containing 60 µg/mL F-Fe_3_O_4_.

Alkaline phosphatase (ALP) staining was performed using the NBT/BCIP ALP staining kit (Beyotime) 7 days after osteogenic induction, and microscopic observation was conducted. The ALP assay kit (Beyotime) was quantified using ALP activity by measuring absorbance at 405 nm.

Calcium mineralization was assessed through Alizarin Red S (ARS) staining (Sigma-Aldrich) 14 days after osteogenic induction. Bound ARS was eluted with 10% cetylpyridinium chloride (Sigma-Aldrich), and the absorbance of the eluate was measured at 562 nm for quantitative analysis.

Quantitative polymerase chain reaction (qRT-PCR) was performed to evaluate osteogenesis-related gene expression, including *ALP* and *RUNX2* (day 7), and *BGLAP* and *SPP1* (day 14). TRIzol reagent (Invitrogen, USA) was used to extract total RNA, and the NanoDrop 8000 spectrophotometer (Thermo Fisher Scientific) was applied to identify RNA concentration. The PrimeScript RT Reagent Kit (Takara, Japan) was further utilized for inverse transcription. The qRT-PCR System (7500 Applied Biosystems, USA) was used for RT-qPCR. Gene expression levels were normalized to *GAPDH* as the internal reference. Table S1 shows primer sequences.

Immunofluorescence staining for ALP and osteocalcin (OCN) was performed on days 7 and 14 to assess the protein expression, respectively. Further, hBMSCs were fixed with paraformaldehyde, permeabilized with Triton X-100, and blocked with bovine serum albumin (Solarbio, China). Samples were incubated overnight at 4℃ with primary antibodies, then with secondary antibodies for 2 h at RT, and stained with DAPI. Fluorescence images were captured utilizing CLSM.

### RNA sequencing (RNA-Seq) and bioinformatics analysis

The total RNA of hBMSCs in OM, F-Fe_3_O_4_, and F-Fe_3_O_4_+SMF groups was extracted using TRIzol after 7 days of osteogenic induction. Three replicates for each sample were sequenced. The Bioanalyzer 2100 platform (Agilent Technologies, USA) was utilized to assess RNA integrity and purity. The analyses were conducted using Novogene Company (Beijing, China). DEGSeq2 software (ver. 1.20.0) was used for differential expression analysis. The adjusted *p*-value of 0.05 and |log2FoldChange| > 0 were set as the thresholds for significantly differential expression. Various analyses, including Gene Ontology (GO), Kyoto Encyclopedia of Genes and Genomes (KEGG), and Gene Set Enrichment Analysis (GSEA), were adopted to identify the gene functional enrichment, screen the differentially expressed genes (DEGs) and pathways, and determine underlying mechanisms.

### Verification of the osteogenic differentiation mechanisms

The medium was replaced for osteogenic induction when hBMSCs reached 70%–80% confluence. The treatments for the PM, OM, F-Fe_3_O_4_, and F-Fe_3_O_4_+SMF groups were as described above. Additional groups were included to verify the role of *SLC2A1*: F-Fe_3_O_4_+SMF+siSLC2A1 (*SLC2A1* knockdown by siRNA), F-Fe_3_O_4_+SMF+siSLC2A1 + ML228 (adding HIF-1 signaling pathway activator [53]), and F-Fe_3_O_4_+SMF+siSLC2A1 + SC79 (adding PI3K/AKT signaling pathway activator [54]) for rescue validation. These three groups were subjected to siRNA transfection before osteogenic induction (the siSLC2A1 sequences were as follows: GACACUAAGUUAUAGUAUAUC [sense], UAUACUAUAACUUAGUGUCUG [antisense]) with Lipofectamine 2000 (Invitrogen). The medium was replaced after transfection. Pathway-blocking groups, including F-Fe_3_O_4_+SMF + PX-478 (adding HIF-1 signaling pathway inhibitor [55]) and F-Fe_3_O_4_+SMF+LY294002 (adding PI3K/AKT signaling pathway inhibitor [56]), were established to validate pathway involvement.

ALP and ARS staining were performed, and followed by quantitative analysis. After 7 days of osteogenic induction, RT-qPCR was performed to assess the mRNA expression level of *RUNX2*, *SLC2A1*, *HIF1A*, and *AKT2*. The procedures were as described above, and Table S1 lists the primer sequences. Furthermore, immunofluorescence staining was performed to assess the protein expression of Runt-related transcription factor 2 (RUNX2), Glucose transporter 1 (GLUT1), Hypoxia-inducible factor 1-alpha (HIF-1α), and Protein Kinase B (AKT). ImageJ was used to quantify the fluorescence intensity of the positive areas in immunofluorescence images.

### In vivo animal experiments

The Laboratory Animal Welfare and Ethics Committee of the Biomedical Ethics Committee at Peking University (LA2020514) approved the animal experiments. Eight-week-old male Sprague–Dawley rats (200–220 g) purchased from Beijing Vital River Laboratory Animal Technology Company (Beijing, China) were used to create the infectious femoral defect model. After intraperitoneal injection of 1% sodium pentobarbital (50 mg/kg) for anesthesia, the hind limbs of rats were shaved and disinfected. The depth of anesthesia was monitored by the loss of the pedal withdrawal reflex. Body temperature was maintained throughout the surgery using a homeothermic heating system. The femoral condyles were exposed via blunt dissection, and cylindrical bone defect models (3 mm in diameter and 3 mm in depth) were developed using an electric drill. The suspension of *S. aureus* (10^6^ CFU/mL) was injected into the defect cavity to induce infection. 36 rats were randomly assigned to four groups: blank (no treatment), GelMA (GelMA hydrogel injection), F-Fe_3_O_4_ (GelMA hydrogel containing 60 µg/mL F-Fe_3_O_4_), and F-Fe_3_O_4_+SMF (GelMA hydrogel containing 60 µg/mL F-Fe_3_O_4_ with SMF exposure). The same GelMA amount were used consistently across the latter three GelMA-used groups, thereby minimizing the influence of GelMA exposure as a potential confounding factor when comparing different treatments. Carprofen was administered for postoperative pain management. For F-Fe_3_O_4_+SMF group, multiple NdFeB permanent magnets were fixed directly beneath the cage floor to provide continuous*tibacterial activity of different magnetic particle group* SMF exposure [57]. The magnetic field strength measured at designated reference points on the cage floor was approximately 100 mT. Because the rats were allowed to move freely in the cage, the actual field experienced at the level of the hind limbs varied with their position relative to the magnets. Therefore, the reported 100 mT should be regarded as a reference field strength rather than a uniform and constant individual exposure level, and the spatial uniformity of the magnetic field across the entire exposure area was not systematically characterized. EFL-Tech (China) supplied the GelMA hydrogel, according to the manufacturer’s instructions.

Three rats from each group were euthanized by an intraperitoneal injection of an overdose of 1% pentobarbital sodium at 150 mg/kg body weight after 1 week, and the major organs (the heart, liver, spleen, lung, and kidney) and the soft tissue surrounding the defects were collected. Sterile swabs were utilized to collect samples from the bone defect area to assess antibacterial performance. The distal femurs, along with the major organs, were removed. Hematoxylin–eosin (H&E) and Giemsa staining were performed on the collected soft tissue around the bone defects to assess antibacterial capacity. H&E staining of the major organs was performed to examine biosafety. At 4 and 8 weeks post-operation, micro-CT scanning was performed on the harvested bone tissue samples employing the NanoVoxel-1000 system (Sanying Precision Instruments, China), and Dragonfly software (Comet Technologies, Canada) was used for image reconstruction and analyses. Parameters, including the new bone volume to total tissue volume ratio (BV/TV), trabecular number (Tb.N), trabecular thickness (Tb.Th), and trabecular separation (Tb.Sp) were calculated. The femur samples were decalcified, dehydrated, embedded, sectioned, and stained. H&E staining and Masson’s trichrome staining results, as well as the immunofluorescence staining results for OCN were observed using the microscope.

### Statistical analysis

The SPSS version 24.0 software (IBM, USA) was used for statistical analysis. All analyses of differences were presented as mean ± standard deviation (SD). The differences among groups were evaluated using one-way analysis of variance (with Tukey’s post hoc test for multiple comparisons) or Student’s t-test. A *p*-value of < 0.05 indicated statistically significant differences.

## Results

### Fabrication and characterization of F-Fe_3_O_4_

**Fig. 1:**
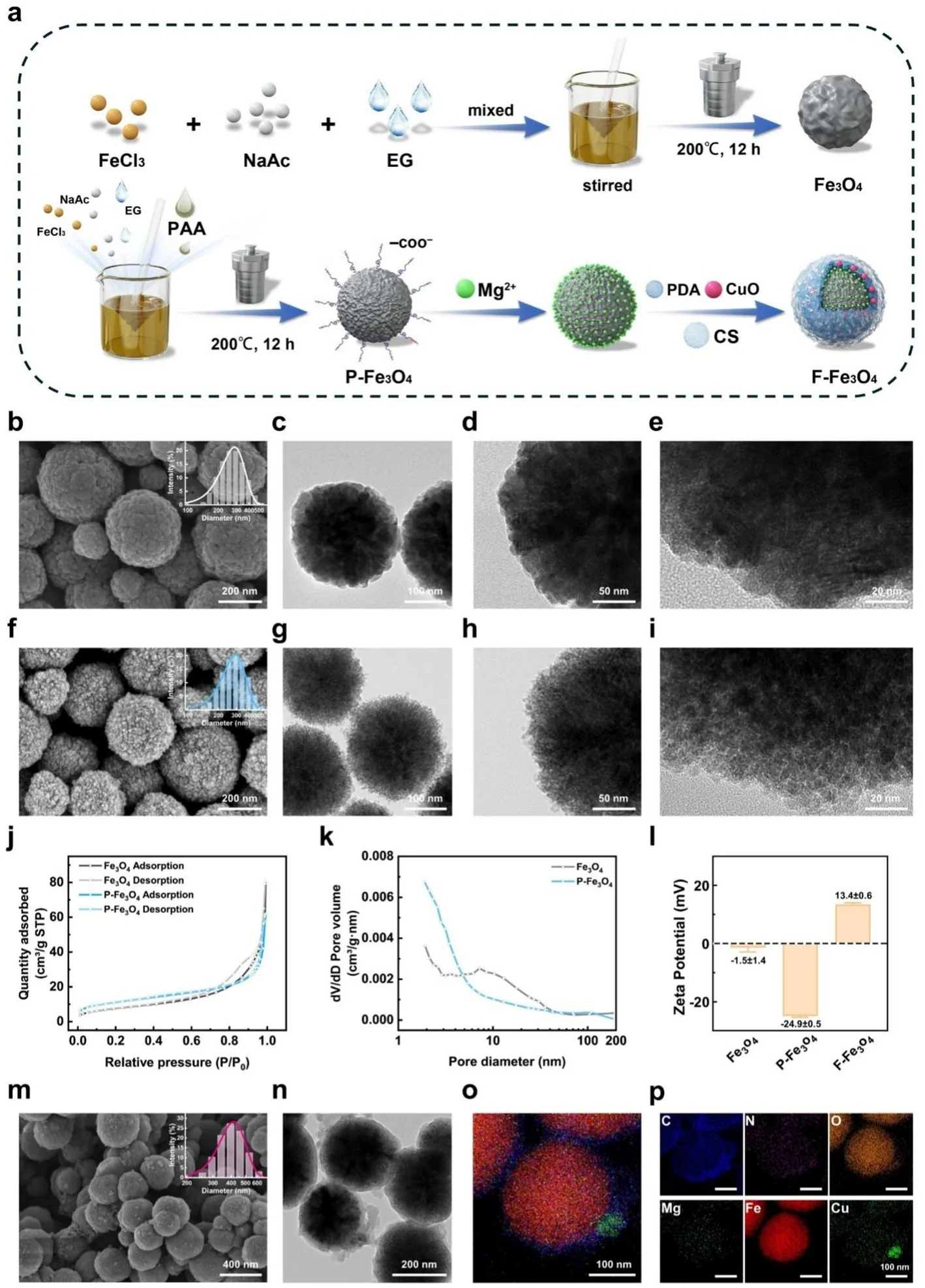
Preparation and characterization of Fe_3_O_4_, P-Fe_3_O_4_, and F-Fe_3_O_4_. **a** Synthesis progress of Fe_3_O_4_, P-Fe_3_O_4_, and F-Fe_3_O_4_; (**b**) SEM image and hydrodynamic diameter distribution of Fe_3_O_4_; (**c**–**e**) TEM images of Fe_3_O_4_; (**f**) SEM image and hydrodynamic diameter distribution of P-Fe_3_O_4_; (**g**–**i**) TEM images of P-Fe_3_O_4_; (**j**) N_2_ adsorption-desorption isotherms of Fe_3_O_4_ and P-Fe_3_O_4_; (**k**) adsorption pore size distribution of Fe_3_O_4_ and P-Fe_3_O_4_ determined by the Barrett-Joyner-Halenda method; (**l**) Zeta potential of Fe_3_O_4_, P-Fe_3_O_4_, and F-Fe_3_O_4_; (**m**) SEM image and hydrodynamic diameter distribution of F-Fe_3_O_4_; (**n**) TEM image of F-Fe_3_O_4_; (**o**) EDS elemental mapping of F-Fe_3_O_4_ (overlay, Mo grid); (**p**) individual elemental mapping of C, N, O, Mg, Fe, and Cu in F-Fe_3_O_4_

**Fig. 2:**
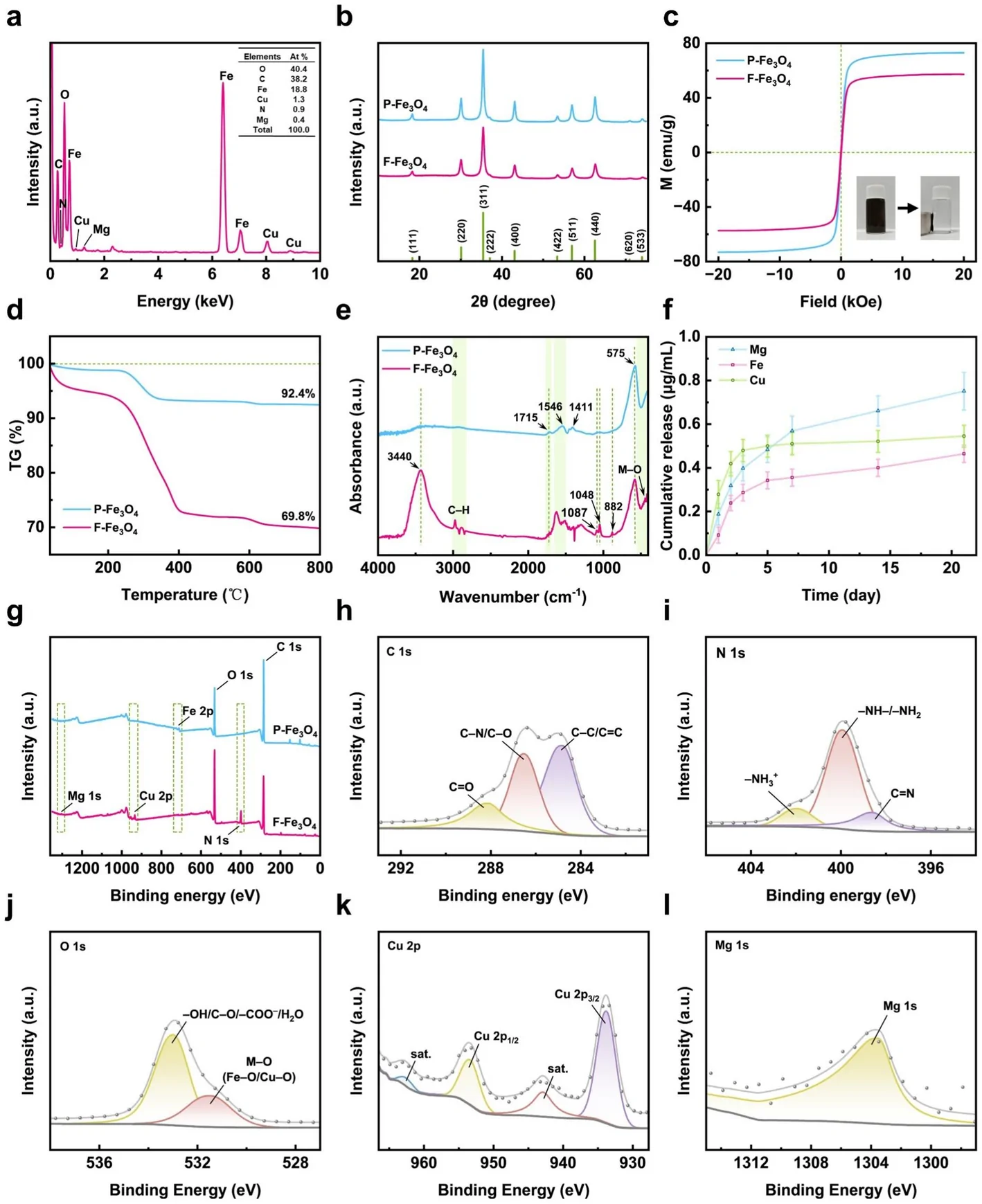
Physicochemical characterization odifferent magnetic particle groups on the biocompatibility, proliferation, migration and Mg 1s

The one-pot solvothermal method was used to synthesized Fe_3_O_4_ and P- Fe_3_O_4_ particles (Fig. 1a). SEM and TEM images revealed spherical morphologies for both Fe_3_O_4_ (Fig. 1b–e) and P-Fe_3_O_4_ (Fig. 1f–i). The inset DLS data indicated comparable size distributions, whereas PAA incorporation caused a notable change in surface morphology. Using the BET method, N_2_ adsorption–desorption isotherms of both samples exhibited distinct hysteresis loops (Fig. 1j) [58]. Further analysis in Table S2 indicated a 42% increase in the specific surface area of P-Fe_3_O_4_ relative to Fe_3_O_4_, along with a markedly smaller average pore diameter. T-plot analysis and pore-size distribution comparisons demonstrated that the micropore area and volume of P-Fe_3_O_4_ exceeded those of Fe_3_O_4_ (Figs. 1k and S1a). These findings indicate that PAA increases specific surface area and refines pore structure, facilitating subsequent surface functionalization [38]. Additionally, zeta potential measurements showed that the surface charge of P-Fe_3_O_4_ was more negative than that of Fe_3_O_4_ (Fig. 1l). Based on these pore and surface differences, P-Fe_3_O_4_ was selected for loading subsequent components, and stepwise modification yielded F-Fe_3_O_4_ with an overall positive surface charge (Fig. 1l). SEM and TEM images showed that F-Fe_3_O_4_ particles were coated with a rough outer shell containing granular protrusions, and DLS analysis revealed a larger particle size for F-Fe_3_O_4_ relative to P-Fe_3_O_4_ (Fig. 1m and n). Additionally, EDS mapping confirmed the presence of C, N, O, Mg, and Cu in F-Fe_3_O_4_, supporting incorporation of Mg^2+^, CuO, and the N-containing organic layer (Fig. 1o and p; Fig. 2a).

XRD patterns revealed that both samples matched the standard magnetite phase (JCPDS, no. 99 − 0073) with identical peak positions (Fig. 2b), confirming crystal phase stability after multistep modification [59]. The magnetite-related peaks of F-Fe_3_O_4_ showed lower intensity and broader peak widths, along with a raised background (Fig. S1b), likely due to amorphous organic surface coatings [60, 61]. Hysteresis loops indicated typical superparamagnetic behavior at RT; the saturation magnetization of P-Fe_3_O_4_ was 73.13 emu/g, whereas that of F-Fe_3_O_4_ was 57.28 emu/g owing to added surface modifiers (Fig. 2c) [62]. The inset images show that F-Fe_3_O_4_ could be magnetically enriched and separated using a magnet, confirming strong magnetic responsiveness and recyclability [59]. TGA results showed that P-Fe_3_O_4_ exhibited 22.6% greater mass loss compared with F-Fe_3_O_4_ at 800 °C, indicating the presence of organic components (Fig. 2d).

In FT-IR spectra (Fig. 2e), the prominent absorption band around 575 cm^− 1^ corresponded to Fe–O stretching of magnetite, confirming the magnetite core’s presence [44]. For P-Fe_3_O_4_, the characteristic PAA bands appeared at 1546 and 1411 cm^− 1^, attributed to asymmetric and symmetric stretching of carboxylate groups (–COO^–^). The observed wavenumber difference of < 200 cm^− 1^ suggested that the coordination between Fe and –COO^−^ in P-Fe_3_O_4_ was dominated by bridging or bidentate binding modes [63, 64]. The band at 1715 cm^− 1^, assigned to C = O stretching of free carboxyl groups, reflected the carboxyl-rich PAA structure that forms a stable negatively charged surface and provides accessible carboxylate sites for subsequent ion adsorption and coordination [38]. After sequential modification, F-Fe_3_O_4_ demonstrated an intensified broad band at 3440 cm^− 1^ from O–H/N–H stretching of PDA and CS. Moreover, C–H stretching bands at 2974, 2889, and 2834 cm^− 1^ indicated increased organic content. Weak absorption bands at 1710–1745 cm^− 1^ were attributed to C = O [46, 65, 66]. The spectra also showed changes in the 1500–1650 cm^− 1^ region at the P-Fe_3_O_4_-Mg^2+^@PDA-CuO stage (Fig. S1c), corresponding to PDA’s aromatic ring and conjugated structures [65]; these features appeared before CS addition, with characteristic CS bands (1087, 1048, and 882 cm^− 1^) intensifying only after the final step [41, 66]. This suggests that the PDA layer formed prior to and persisted after CS coating. In the low-wavenumber region, besides the Fe–O band, an additional absorption band appeared at 407–445 cm^− 1^, consistent with metal–oxygen lattice vibrations and indicating successful CuO incorporation [67]. The ICP analysis demonstrated stable Fe and Mg release, whereas Cu displayed a higher initial release rate that gradually stabilized (Fig. 2f). In the XPS-detected wide-scan spectrum (Fig. 2g), C, N, O, Fe, Cu, and Mg signals were present in F-Fe_3_O_4_, confirming successful integration of organic layers and metal species. Compared with P-Fe_3_O_4_, the Fe signal in F-Fe_3_O_4_ was markedly attenuated, reflecting shielding of the core by outer organic coatings [68]. Specifically, the high-resolution C 1s spectrum of F-Fe_3_O_4_ (Fig. 2h) was deconvoluted into peaks at 284.8, 286.5, and 288.13 eV, corresponding to C–C/C = C, C–N/C–O, and C = O bonds, respectively, indicating the presence of organic functionalities. The N 1s spectrum (Fig. 2i) displayed peaks at 398.57, 399.92, and 401.94 eV, which were assigned to C = N, –NH–/–NH_2_, and –NH_3_^+^, respectively, confirming the existence of the N-containing organic layer. The peaks of the O 1s spectrum (Fig. 2j) at 531.45 and 532.99 eV were attributed to metal–oxygen bonds (Fe–O and Cu–O) and organic oxygen-containing groups, respectively [69–72]. Although the Fe signal was weak in F-Fe_3_O_4_, the Fe 2p_3/2_ and Fe 2p_1/2_ peaks at 710.85 and 724 eV, respectively, were detected at the intermediate P-Fe_3_O_4_-Mg^2+^@PDA-CuO stage (Fig.S1d and e) [73]. The Cu 2p spectrum showed main peaks at 933.8 and 953.38 eV with satellite peaks at 942.92 and 962.49 eV (Fig. 2k) confirmed Cu^2+^ [74]. As shown in Fig. 2l, the Mg 1s peak at 1303.54 eV verified Mg-related species [75]. Collectively, these results confirm successful fabrication of F-Fe_3_O_4_.

### Biocompatibility assessment of F-Fe_3_O_4_

**Fig. 3:**
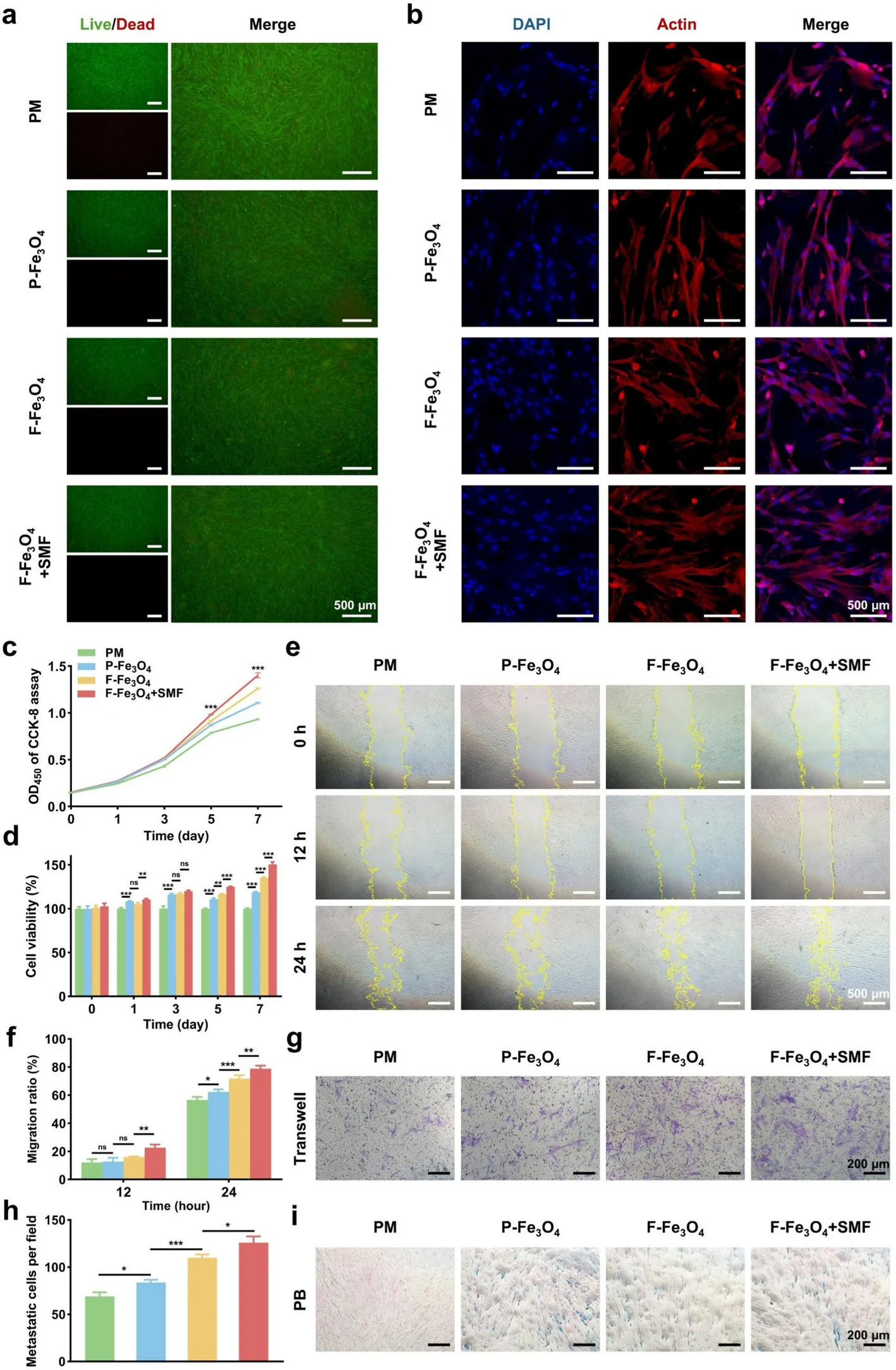
Effects of different magnetic particle groups on the biocompatibility, proliferation, migration and Fe^3+^ internalization of hBMSCs in vitro. **a** Fluorescence images of live/dead staining; (**b**) CLSM images of cytoskeletal staining; (**c**) CCK-8 assay of cell proliferation; (**d**) analysis of cell viability; (**e**) optical images of scratch assay; (**f**) quantitative results of scratch assay; (**g**) optical images of Transwell assay; (**h**) quantitative results of Transwell assay; (**i**) optical images of Prussian blue staining for intracellular iron deposition. Data throughout are means ± SD (n ≥ 3). *p < 0.05; **p < 0.01; ***p < 0.001; ns: not significant, *p* > 0.05

Live/dead staining showed that nearly all cells in PM, P-Fe_3_O_4_, F-Fe_3_O_4_, and F-Fe_3_O_4_+SMF groups remained viable (green), indicating that the magnetic particles neither induced apoptosis nor caused cytotoxic damage (Fig. 3a). Cytoskeletal staining revealed that cells in all groups adhered and spread effectively, exhibiting well-organized F-actin filaments and intact nuclei (Fig. 3b). Cells in the F-Fe_3_O_4_+SMF group displayed more pronounced protrusions and extended pseudopodia, implying enhanced adhesion and spreading capacity. Additionally, cell proliferation was evaluated using the CCK-8 assay on days 0, 1, 3, 5, and 7. As shown in Fig. 3c and d, OD values increased progressively in all groups, reflecting sustained hBMSC proliferation. Notably, the F-Fe_3_O_4_+SMF group exhibited greater cell viability (*p* < 0.001), indicating that F-Fe_3_O_4_ combined with SMF further promoted proliferation.

The migratory ability of hBMSCs was assessed using scratch and Transwell assays. Scratch assay results showed that the F-Fe_3_O_4_+SMF group achieved the greatest wound closure (*p* < 0.01), followed by the F-Fe_3_O_4_ group (Fig. 3e and f). In the Transwell assay, after 24 h of coculture, more transmigrated cells were observed in the F-Fe_3_O_4_ and F-Fe_3_O_4_+SMF groups (Fig. 3g and h), with the F-Fe_3_O_4_+SMF group showing a statistically significant increase compared with the other groups (*p* < 0.05). Prussian blue staining revealed blue precipitates (Prussian blue–positive) within cells in the P-Fe_3_O_4_, F-Fe_3_O_4_, and F-Fe_3_O_4_+SMF groups, confirming Fe^3+^ internalization (Fig. 3i).

These findings indicate that F-Fe_3_O_4_ exhibits favorable biocompatibility and promotes hBMSC proliferation, adhesion, and migration. These promotive effects were further amplified under SMF exposure, highlighting its potential benefits for tissue regeneration.

### In vitro antibacterial activity of F-Fe_3_O_4_

**Fig. 4:**
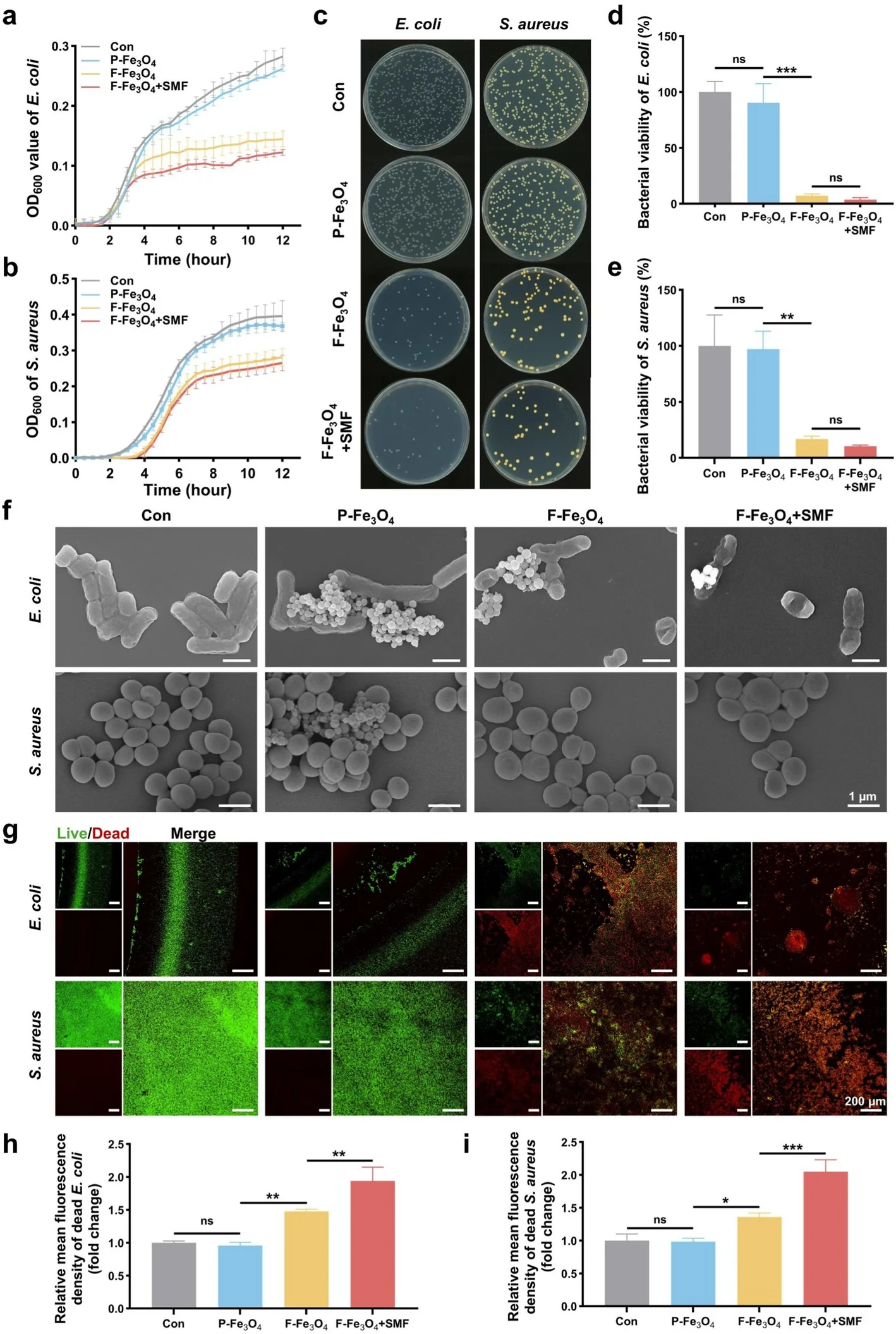
In vitro antibacterial activity of different magnetic particle groups. **a** The growth curve of *E. coli* within 12 h; (**b**) the growth curve of *S. aureus* within 12 h; (**c**) images of bacterial colonies on agar plates (*E. coli *and *S. aureus*); (**d**) bacterial viability of *E. coli*; (**e**) bacterial viability of *S. aureus*; (**f**) SEM images of bacterial morphology; (**g**) CLSM images of live/dead bacterial staining; (**h**) quantitative analysis of the fluorescence intensity of the live/dead staining for *E. coli* and (**i**) *S. aureus*. Data throughout are means ± SD (n ≥ 3). *p < 0.05; **p < 0.01; ***p < 0.001; ns: not significant, *p* > 0.05

To evaluate the antibacterial efficacy of F‑Fe_3_O_4_, representative gram‑negative bacteria (*E. coli*), gram‑positive bacteria (*S. aureus*), and a common oral pathogen (*E. faecalis*) associated with periapical periodontitis were selected. Bacterial growth curves for *E. coli*, *S. aureus*, and *E. faecalis* demonstrated continued proliferation within 12 h in the Con and P‑Fe_3_O_4_ groups. In contrast, the F‑Fe_3_O_4_ and F-Fe_3_O_4_+SMF groups exhibited obviously inhibited bacterial growth, with the latter showing stronger suppression, as reflected by its flatter growth curve (Fig. 4a and b; Fig. S2a). Consistently, colony counts in the F-Fe_3_O_4_ and F-Fe_3_O_4_+SMF groups were significantly reduced compared with the Con and P-Fe_3_O_4_ groups (*p* < 0.01) (Fig. 4c–e; Fig. S2b and c). Specifically, the viability of *E. coli*, *S. aureus*, and *E. faecalis* was approximately 7.19%, 16.95%, and 16.55%, respectively, in the F-Fe_3_O_4_ group and 3.78%, 10.43%, and 3.99%, respectively, in the F-Fe_3_O_4_+SMF group (Fig. 4d and e; Fig. S2c). SEM images showed that bacteria in the Con and P-Fe_3_O_4_ groups retained intact morphology and smooth surfaces (Figs. 4f and S2d). In contrast, bacteria in the F-Fe_3_O_4_ and F-Fe_3_O_4_+SMF groups displayed roughened, shrunken surfaces and morphological deformation. Live/dead staining showed that most bacteria in the F-Fe_3_O_4_ and F-Fe_3_O_4_+SMF groups were dead, whereas those in the Con and P-Fe_3_O_4_ groups remained viable (Figs. 4g and S2e). Further quantitative analysis revealed that the fluorescence intensities of dead bacteria in the F-Fe_3_O_4_ and F-Fe_3_O_4_+SMF groups were significantly higher than those in the Con and P-Fe_3_O_4_ groups (*p* < 0.05) (Figs. 4h and i, Fig. S2f). Moreover, in the F-Fe_3_O_4_+SMF group, the fluorescence intensities of dead *E. coli* and *S. aureus* were higher than those in the F-Fe_3_O_4_ group (*p* < 0.01). Therefore, the F-Fe_3_O_4_ magnetic composite particles exhibited good antibacterial activity, and SMF showed a tendency to enhance the antibacterial performance of F-Fe_3_O_4_.

### In vitro osteogenic differentiation-promoting effects of F-Fe_3_O_4_ on hBMSCs

**Fig. 5:**
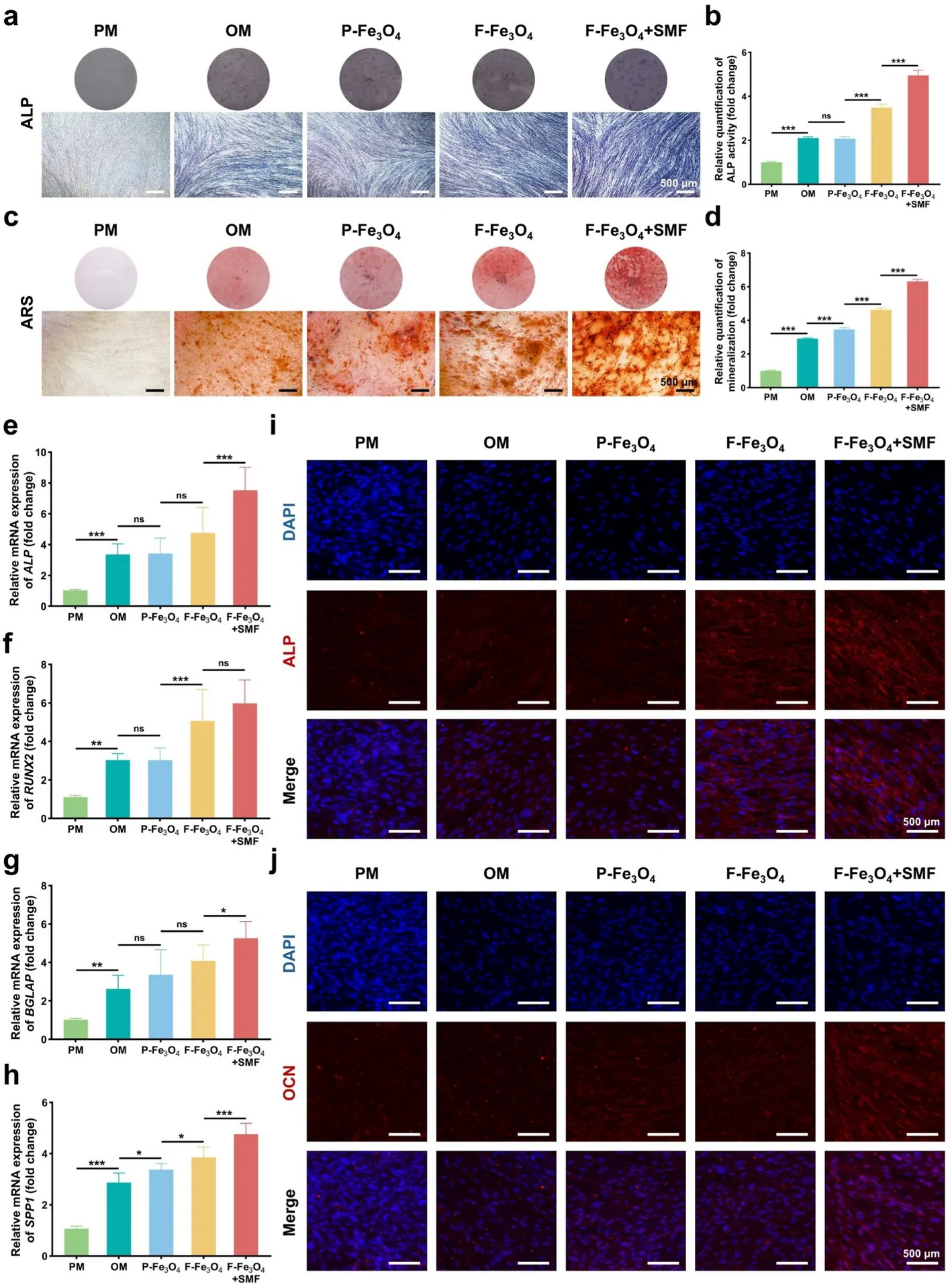
In vitro osteogenic differentiation-promoting effects of different magnetic particle groups on hBMSCs. **a** ALP staining and (**b**) quantitative analysis of ALP activity; (**c**) ARS staining and (**d**) quantitative analysis of mineralization; (**e–h**) qRT-PCR analysis of osteogenic marker genes: *ALP*, *RUNX2*, *BGLAP*, and *SPP1*; (**i**, **j**) immunofluorescence staining images of ALP (**i**) and OCN (**j**). Data throughout are means ± SD (n ≥ 3). *p < 0.05; **p < 0.01; ***p < 0.001; ns: not significant, *p* > 0.05

After 7 days of osteogenic induction, ALP staining and activity assays showed that the F-Fe_3_O_4_+SMF group exhibited the strongest staining (*p* < 0.001), followed by the F-Fe_3_O_4_ group (Fig. 5a and b). After 14 days, ARS staining showed a similar pattern (Fig. 5c). The F-Fe_3_O_4_+SMF group produced the greatest calcium deposition, reflecting superior osteogenic differentiation. Quantification of dissolved mineralized nodules further supported this observation (*p* < 0.001) (Fig. 5d).

To examine the osteogenic differentiation-promoting ability of F-Fe_3_O_4_ in hBMSCs at the transcriptional level, the mRNA expression of four osteogenesis-related genes was analyzed via qRT-PCR at defined induction stages: *ALP* and *RUNX2* on day 7, and *BGLAP* and *SPP1* on day 14. Results revealed that hBMSCs in the F-Fe_3_O_4_+SMF group expressed the highest mRNA levels of *ALP* and *BGLAP* (*p* < 0.05), whereas *RUNX2* and *SPP1* expression levels were significantly upregulated in the F-Fe_3_O_4_ and F-Fe_3_O_4_+SMF groups (*p* < 0.05), with further enhancement in the F-Fe_3_O_4_+SMF group (Fig. 5e–h). These data indicate that F-Fe_3_O_4_ promotes the osteogenic differentiation of hBMSCs, and SMF addition further elevates the expression of osteogenesis-related genes, increasing early and mid‑to‑late stage osteogenic marker levels. Additionally, after 7 days of osteogenic induction, ALP immunofluorescence staining showed strong fluorescence in the F-Fe_3_O_4_ and F-Fe_3_O_4_+SMF groups, confirming activation of early osteogenesis (Fig. 5i). After 14 days, OCN immunofluorescence staining revealed obvious OCN signals in the F-Fe_3_O_4_+SMF group, indicating promotion of the mature osteogenic phenotype and mineralization‑related processes (Fig. 5j).

Overall, these results demonstrate that F-Fe_3_O_4_ promotes hBMSC osteogenic differentiation from early to mid‑to-late stages and accelerates mineral maturation, while the addition of SMF provides further enhancement.

### In vivo antibacterial effects of F-Fe_3_O_4_

**Fig. 6:**
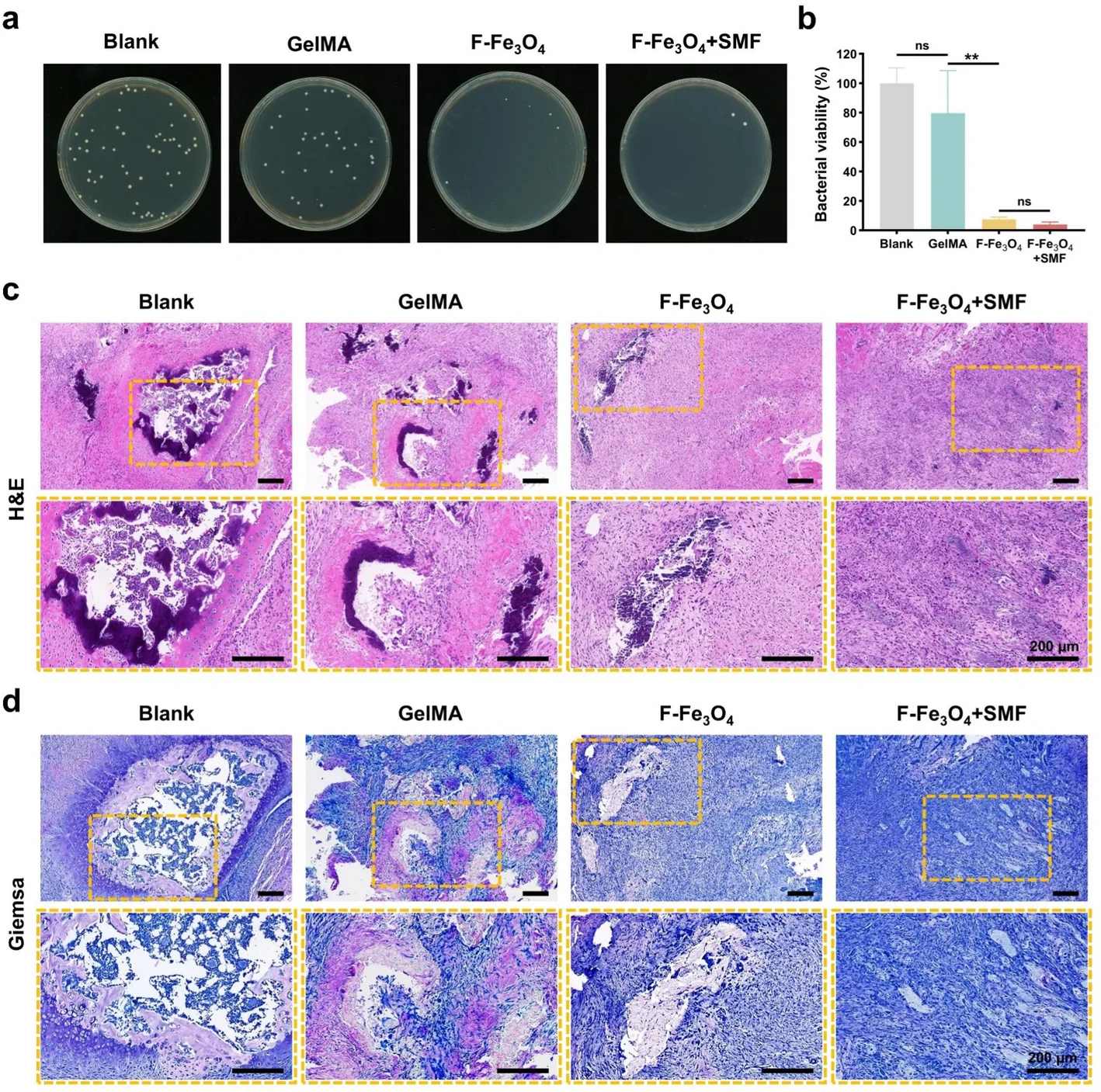
In vivo antibacterial activity of different magnetic particle groups. **a** Images of bacterial colonies on agar plates; (**b**) analysis of bacterial viability; (**c**) H&E staining, and (**d**) Giemsa staining images of peri-defect soft tissues. Data throughout are means ± SD (n ≥ 3). *p < 0.05; **p < 0.01; ***p < 0.001; ns: not significant, *p* > 0.05

An infected femoral defect model was established to evaluate the magnetic nanoparticles’ in vivo performance. One week after operation, three rats per group were randomly selected for antibacterial assessment. Colony counts revealed bacterial survival rates of ~ 7.46% in the F-Fe_3_O_4_ group and ~ 3.98% in the F-Fe_3_O_4_+SMF group, indicating that both treatments achieved good early-stage in vivo antibacterial activity (*p* < 0.01) (Fig. 6a and b).

Histological staining of peri-defect soft tissue, including H&E staining for inflammatory infiltration and Giemsa staining for bacterial detection, was performed to assess infection and inflammation. H&E staining showed prominent inflammatory cell infiltration in the Blank and GelMA groups, reflecting a pronounced inflammatory response (Fig. 6c). In contrast, inflammation decreased in the F-Fe_3_O_4_ and F-Fe_3_O_4_+SMF groups, with the lowest level observed in the F-Fe_3_O_4_+SMF group. Giemsa staining corroborated this pattern, showing substantial bacterial presence in the Blank and GelMA groups, whereas the F-Fe_3_O_4_ group exhibited reduced bacterial load while the F-Fe_3_O_4_+SMF group displayed minimal colonization (Fig. 6d). These findings, consistent with the in vitro antibacterial results, indicate that F-Fe_3_O_4_+SMF can inhibit early bacterial growth in vivo, enhances local resistance to infection, and mitigates inflammation.

### In vivo osteogenic effects and biosafety of F-Fe_3_O_4_

**Fig. 7:**
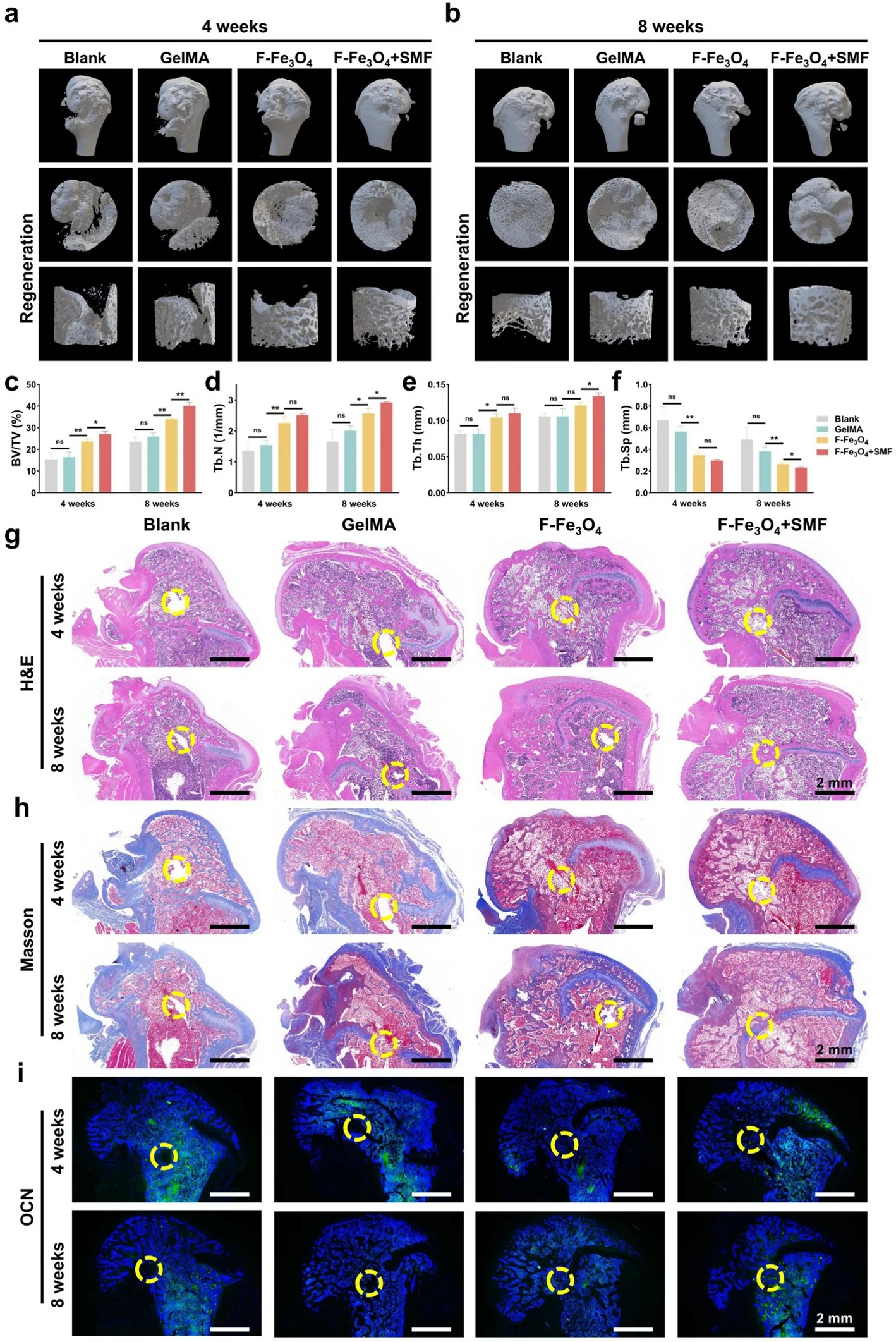
In vivo bone regeneration of different magnetic particle groups. **a**, **b** Reconstructed micro-CT images and ROI views from different angles at 4 weeks (**a**) and 8 weeks (**b**); (**c**–**f**) quantification of bone morphometric parameters: the new bone volume to total tissue volume ratio (BV/TV), trabecular bone number (Tb.N), trabecular thickness (Tb.Th), and trabecular separation (Tb.Sp); (**g**) H&E staining, (**h**) Masson’s trichrome staining and (**i**) immunofluorescence staining of OCN of bone tissues at 4 and 8 weeks. Data throughout are means ± SD (n ≥ 5). *p < 0.05; **p < 0.01; ***p < 0.001; ns: not significant, *p* > 0.05

Samples were harvested at 4 and 8 weeks post-operation, and osteogenesis at the defect site was analyzed using micro-CT reconstruction. At 4 weeks, reparative tissue was present in all groups; however, both the quantity and quality of new bone varied. In the Blank and GelMA groups, newly formed bone appeared discontinuous, with irregular margins and visible gaps, failing to fully bridge the defect area (Fig. 7a). In contrast, new bone in the F-Fe_3_O_4_ and F-Fe_3_O_4_+SMF groups was more continuous and largely covered the defect region. At 8 weeks, continuous bone formed in the Blank and GelMA groups, covering the defect, but surface irregularities remained and overall bone volume was low (Fig. 7b). In the F-Fe_3_O_4_ and F-Fe_3_O_4_+SMF groups, bone formation further increased, and defects were no longer evident in the F-Fe_3_O_4_+SMF group. Quantitative analysis showed that the F-Fe_3_O_4_+SMF group exhibited the highest BV/TV (*p* < 0.05), followed by the F-Fe_3_O_4_ group (Fig. 7c). Tb.N and Tb.Th exhibited similar trends (Fig. 7d and e). Tb.Sp was significantly greater in the Blank and GelMA groups (*p* < 0.01) (Fig. 7f), indicating inferior bone quality. Histopathological changes were further assessed via H&E and Masson’s trichrome staining. H&E staining revealed prominent inflammatory cell infiltration and disorganized bone tissue architecture in the Blank and GelMA groups, highlighting limited regeneration (Fig. 7g). In comparison, the F-Fe_3_O_4_ and F-Fe_3_O_4_+SMF groups demonstrated more abundant, well-organized bone tissue with substantial new formation. Masson staining showed denser and more mature collagen fibers in the F-Fe_3_O_4_+SMF group relative to the other groups (Fig. 7h). Besides, immunofluorescence staining revealed more OCN-positive areas in the F-Fe_3_O_4_ and F-Fe_3_O_4_+SMF groups compared to the Blank and GelMA groups (Fig. 7i). Notably, the fluorescence intensity was strongest in the F-Fe_3_O_4_+SMF group, indicating substantial new bone formation. Collectively, these in vivo data indicate that combining F-Fe_3_O_4_ with SMF effectively promotes bone regeneration and infectious bone defect repair.

Short-term in vivo biosafety was also assessed via H&E staining of major organs (the heart, liver, spleen, lung, and kidney) harvested at 1, 4, and 8 weeks to examine systemic inflammatory responses. No evident pathological changes or morphological abnormalities were observed in these tissues (Figs. S3–5), and no adverse effects or signs of acute systemic toxicity were detected, indicating that magnetic particles at this dose exhibit favorable in vivo biosafety.

### Potential osteogenic differentiation mechanism of F-Fe_3_O_4_ on hBMSCs

**Fig. 8:**
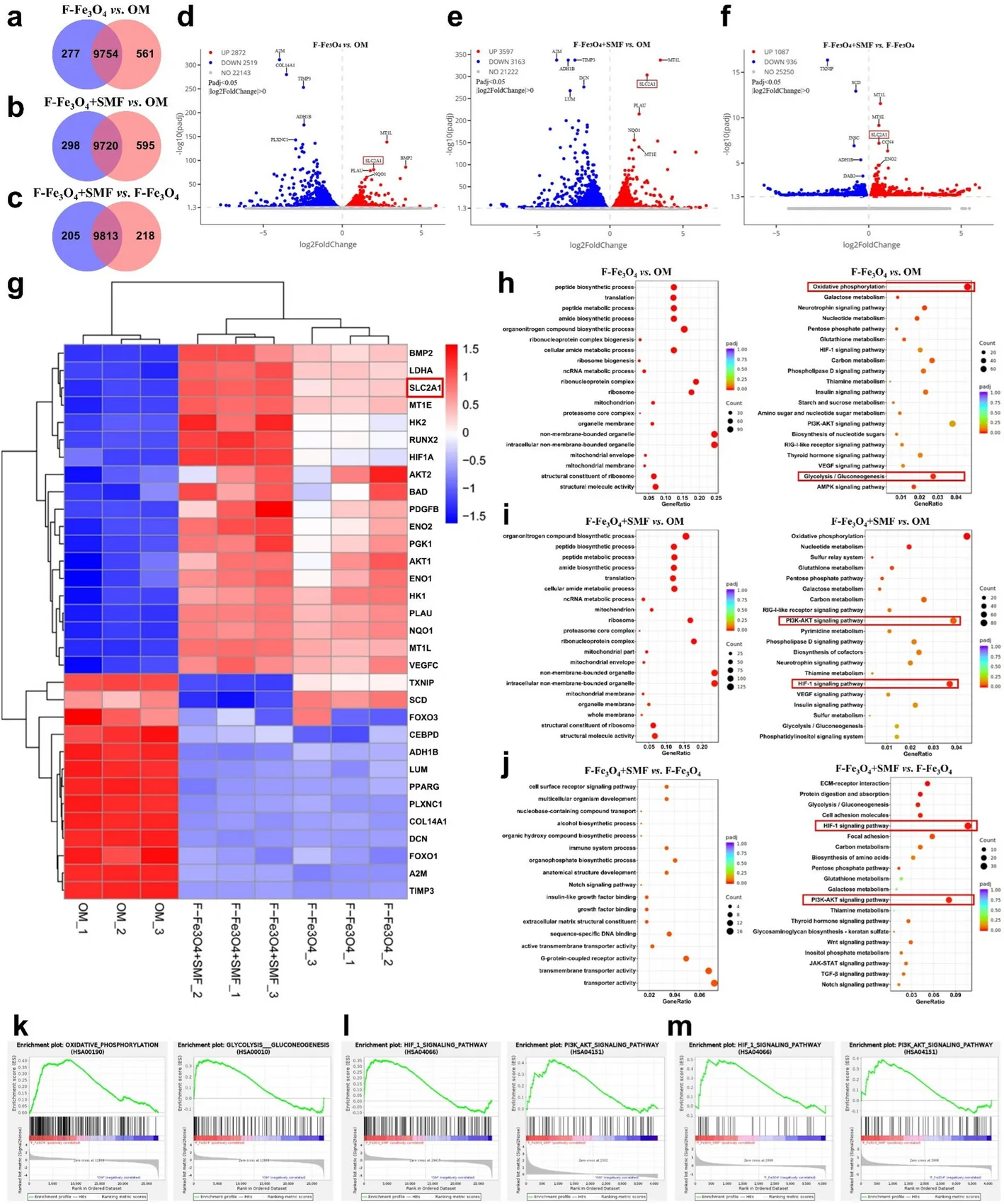
RNA-Seq analysis of the osteogenic differentiation mechanism of F-Fe_3_O_4_+SMF on hBMSCs in vitro. **a**–**c** Venn diagrams displaying the overlap of DEGs among the indicated comparisons: F-Fe_3_O_4_ vs. OM, F-Fe_3_O_4_+SMF vs. OM, and F-Fe_3_O_4_+SMF vs. F-Fe_3_O_4_; (**d**–**f**) volcano plots of DEGs for each comparison (red, upregulated; blue, downregulated); (**g**) Local clustering heatmap of DEGs; (**h**–**j**) GO (left) and KEGG (right) enrichment bubble plots for DEGs in the corresponding comparisons; (**k**–**m**) GSEA enrichment plots for the corresponding comparisons

After 7 days of osteogenesis induction, hBMSCs from the OM, F-Fe_3_O_4_, and F-Fe_3_O_4_+SMF groups were subjected to RNA-seq analysis. The Venn diagram in Fig. 8a–c shows DEGs across various comparisons. In Fig. 8d–f, volcano plots illustrate the magnitude and statistical significance of expression changes, depicting the distributions of upregulated and downregulated genes. Heatmap clustering indicated that the F-Fe_3_O_4_+SMF group was clearly separated from the OM and F-Fe_3_O_4_ groups, with a distinct cluster of stably expressed genes (Fig. 8g). In the F-Fe_3_O_4_+SMF group, upregulated genes, including *SLC2A1* and *HK2*, were primarily associated with glucose metabolism and glycolysis, and osteogenesis-related genes, such as *RUNX2*, demonstrated an increasing trend. Genes involved in angiogenesis, stress response, and repair were also expressed at relatively high levels. Based on the results of pairwise comparison volcano plots and the local clustering heatmap, *SLC2A1* was selected as the key candidate gene for further verification because it showed significant changes across all cases. GO and KEGG analyses of DEGs indicated that the F-Fe_3_O_4_ and F-Fe_3_O_4_+SMF groups were enriched in energy metabolism pathways, including oxidative phosphorylation, whereas F-Fe_3_O_4_+SMF showed significant enrichment in the HIF-1 and PI3K/AKT pathways (Fig. 8h–j). GSEA corroborated the pathways identified via DEG analysis (Fig. 8k–m). Collectively, these findings suggest that SMF combined with F-Fe_3_O_4_ may enhance osteogenesis by promoting *SLC2A1*-mediated glucose uptake and metabolism and modulating the HIF-1 and PI3K/AKT signaling pathways [76].

**Fig. 9:**
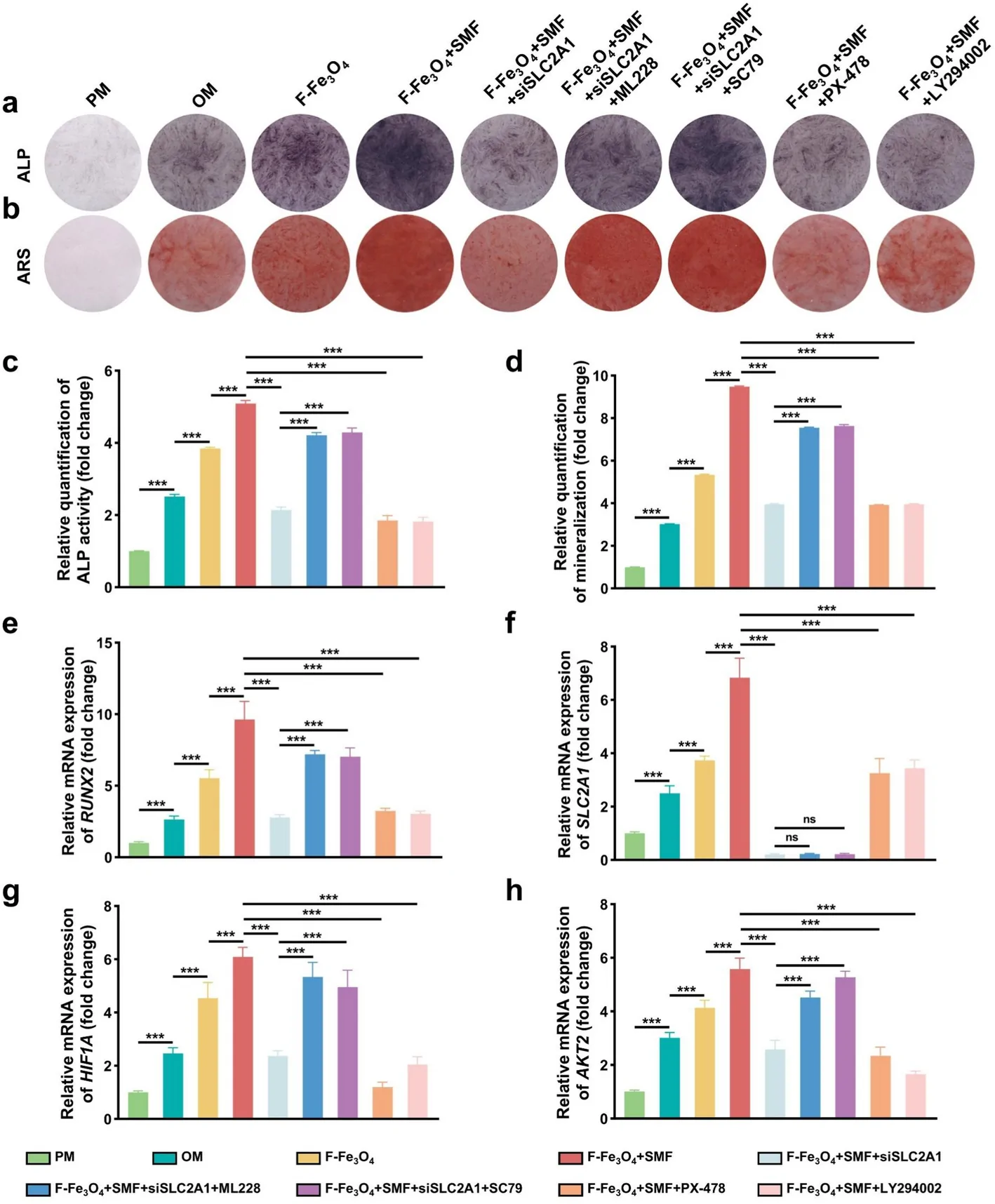
Verification of the osteogenic differentiation mechanism of F-Fe_3_O_4_+SMF on hBMSCs in vitro. **a** ALP staining; (**b**) ARS staining; (**c**) quantification of ALP activity; (**d**) quantification of mineralization; (**e**–**h**) qRT-PCR analysis of *RUNX2*, *SLC2A1*, *HIF1A*, and *AKT2*. Data throughout are means ± SD (n ≥ 3). *p < 0.05; **p < 0.01; ***p < 0.001; ns: not significant, *p* > 0.05

Based on ALP and ARS staining, the F-Fe_3_O_4_+SMF group exhibited the strongest staining, whereas the F-Fe_3_O_4_+SMF+siSLC2A1 group and both pathway inhibition groups showed significant reductions (Fig. 9a and b). Further quantitative analysis revealed that *SLC2A1* knockdown significantly reduced ALP activity and mineralized nodule formation in the F-Fe_3_O_4_+SMF+siSLC2A1 group (*p* < 0.001), underscoring the essential role of *SLC2A1* in F-Fe_3_O_4_ plus SMF–induced osteogenesis (Fig. 9c and d). The differences observed in rescue and pathway blockade experiments further highlighted the critical involvement of HIF-1 and PI3K/AKT signaling in mediating osteogenesis under combined F-Fe_3_O_4_ and SMF treatment.

**Fig. 10:**
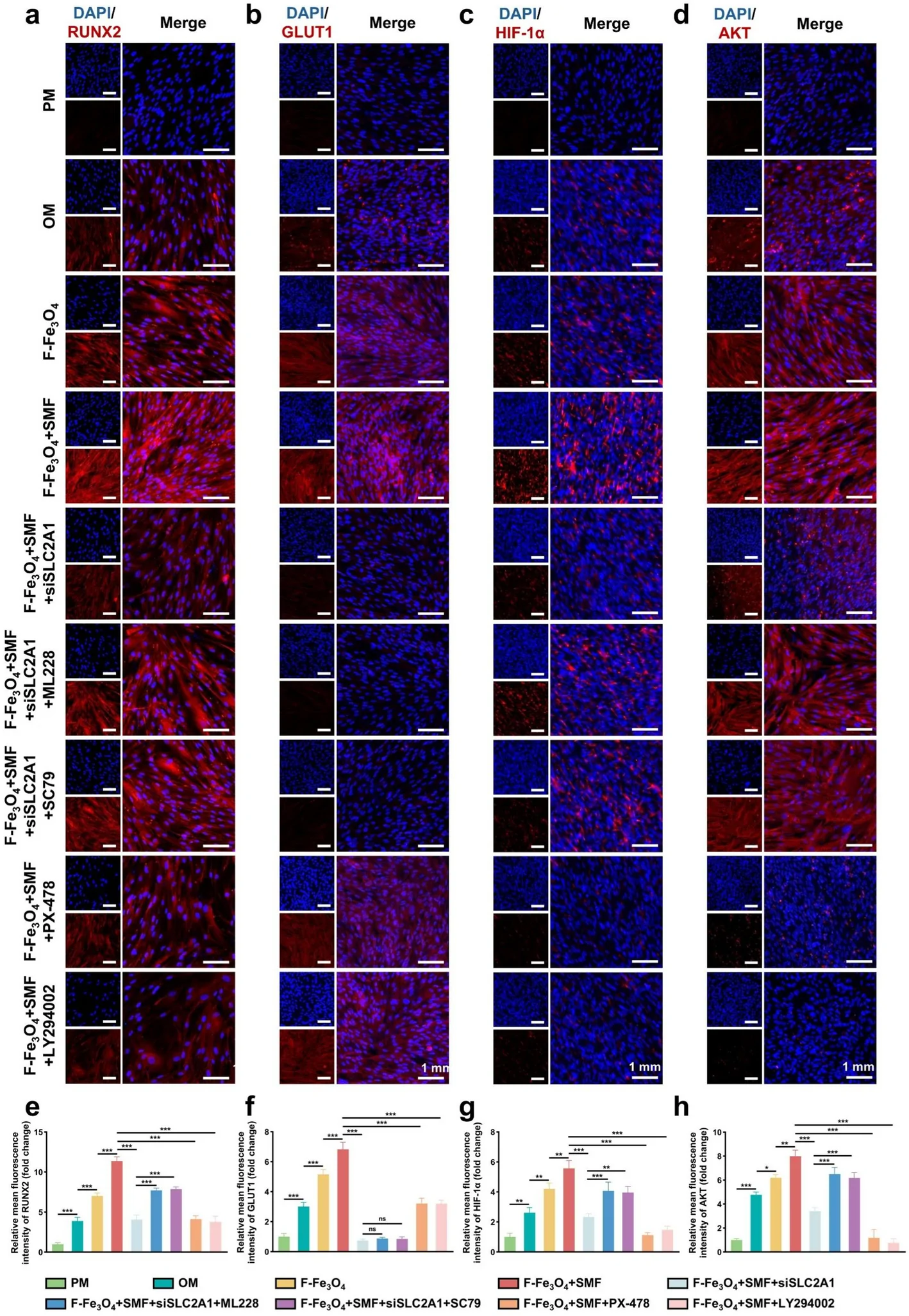
Verification of the osteogenic differentiation mechanism of F-Fe_3_O_4_+SMF on hBMSCs in vitro. **a**–**d** Immunofluorescence staining images of RUNX2, GLUT1, HIF-1α, and AKT; (**e**–**h**) quantitative analysis of the corresponding fluorescence intensity. Data throughout are means ± SD (n ≥ 3). *p < 0.05; **p < 0.01; ***p < 0.001; ns: not significant, *p* > 0.05

RT-qPCR results showed that hBMSCs in the F-Fe_3_O_4_+SMF group exhibited significantly higher expression levels of *RUNX2*, *SLC2A1*, *HIF1A*, and *AKT2* compared with other groups (*p* < 0.001), indicating that the combined F-Fe_3_O_4_ and SMF treatment significantly upregulated these genes and promoted osteogenic differentiation (Fig. 9e–h). In the F-Fe_3_O_4_+SMF+siSLC2A1 group, the expression levels of all genes were significantly reduced (*p* < 0.001), with the most pronounced decrease observed for *SLC2A1*. In the rescue groups, *RUNX2*, *AKT2*, and *HIF1A* expression levels were significantly restored (*p* < 0.001), whereas *SLC2A1* expression remained suppressed due to gene knockdown. These findings confirm the pivotal role of *SLC2A1* in osteogenesis, demonstrating that its silencing suppresses HIF-1 and PI3K/AKT signaling, thereby impairing cellular metabolism and osteogenic differentiation. Moreover, both pathway inhibitor–treated groups showed significantly lower expression levels for all four genes relative to the F-Fe_3_O_4_+SMF group (*p* < 0.001), further supporting the essential roles of HIF-1 and PI3K/AKT signaling in metabolic regulation and osteogenesis. Immunofluorescence staining of RUNX2, GLUT1, HIF-1α, and AKT (Fig. 10a–d), together with quantitative fluorescence analysis (Fig. 10e–h), corroborated these results.

In summary, *SLC2A1* plays an essential role in cellular metabolism and osteogenic differentiation of hBMSCs. Further, the inhibition and activation of HIF-1 and PI3K/AKT signaling pathways regulate the expression of *SLC2A1* and osteogenic differentiation. Combined SMF and F-Fe_3_O_4_ treatment in hBMSCs upregulates *SLC2A1*, thereby activating the HIF-1 and PI3K/AKT signaling pathways, improving cellular metabolism and energy supply, and subsequently upregulating RUNX2 to ultimately accelerate osteogenic differentiation.

## Discussion

Infectious bone defects involve concurrent infection and bone injury; because severe infection can cause nonunion and progressive osteolysis, prioritizing infection control is critical for successful repair. Clinically, antibiotics are limited by resistance and adverse effects, debridement is invasive and carries complication risks, and bone grafts or scaffolds may fail or become reinfected, sometimes requiring secondary surgery. Therefore, material-based strategies capable of achieving effective antibacterial activity and bone regeneration without damaging healthy tissue are urgently required [3, 77]. This study addresses the therapeutic challenge of infectious bone defects, in which bacterial control and tissue regeneration are tightly linked and difficult to separate. We developed a magnetic particle–based system, F-Fe_3_O_4_, integrating SMF responsiveness with multilayered antibacterial and prohealing modules to coordinate multiple functions on a single platform.

During F-Fe_3_O_4_ fabrication, P-Fe_3_O_4_ served as the base structure; incorporation of PAA increased accessible surface area, altered surface charge, and facilitated Mg^2+^ modification (Fig. 1). Although PAA has been widely applied for surface modification, the resulting surface morphology and pore size have varied across reports [38, 43, 44]. For example, Brito et al. [39] functionalized PAA-coated iron oxide nanoparticles with a Pt(_IV_) prodrug for cancer therapy, where the Fe_3_O_4_@PAA nanostructure exhibited a stronger negative charge. Such discrepancies likely stem from differences in preparation methods and reagent ratios. Mg^2+^ can adsorb onto anionic PAA sites, and studies have shown that Mg^2+^ bridges anionic groups, strengthening intermolecular interactions [46, 78]. Research on bisphosphonate–Mg^2+^ self-assembly systems has indicated that Mg^2+^ functions as a structural linker, highlighting its possible multifunctional role in this study [79]. PDA, a mussel-inspired coating, forms adhesive layers on diverse inorganic and organic substrates and is frequently used as an intermediate coupling layer for secondary functionalization in multilayer systems [42, 80]. In F-Fe_3_O_4_, the PDA layer was situated between the magnetic core and outer functional components, providing anchoring sites for CuO loading and CS shell construction. ICP analysis showed rapid Cu ion released initially, followed by a slower release that gradually plateaued (Fig. 2f). This pattern likely reflects early dissolution of surface-bound or weakly bound Cu, with later stages influenced by metal ion chelation via PDA and CS functional groups [81, 82]. In contrast, Mg^2+^, in ionic form, displayed a steadier, gradually increasing release profile [83]. The relatively low and slow Fe release likely resulted from magnetic core stability and limited iron ion migration [81, 84]. These results suggest that the observed intrinsic ion release behavior of F-Fe_3_O_4_ under physiological conditions can be associated with diffusion and physicochemical interactions within the PAA/PDA/CuO/CS multilayer structure.

In this study, the biological performance of F-Fe_3_O_4_ (including biocompatibility, antibacterial activity, osteogenic potential, and biosafety) was evaluated, with an emphasis on this composite multifunctional platform’s effect in infected bone repair, as well as its performance when combined with SMF. Although P-Fe_3_O_4_ provides magnetic responsiveness, it is not intended to independently achieve antibacterial or osteo-regenerative functions. Therefore, surface components were sequentially introduced to endow complementary antibacterial, osteogenic, and interfacial properties, forming a complete therapeutic system before evaluating the additional regulatory effect of SMF. Accordingly, the differences between P-Fe_3_O_4_ and F-Fe_3_O_4_ should be interpreted as the integrated effect of the sequential surface functionalization rather than the isolated contribution of Mg^2+^, PDA, CuO, CS, or other individual components. Furthermore, the subsequent comparison between F-Fe_3_O_4_ and F-Fe_3_O_4_+SMF was designed to evaluate the additional effect of SMF which may further enhance the intrinsic activity of the material. Importantly, SMF was not the sole therapeutic factor; rather, F-Fe_3_O_4_ itself possesses therapeutic capabilities in the absence of SMF, whereas SMF provides an additional modulatory or enhancing effect. Thus, the present results demonstrate the combined effect of hierarchical functionalization and SMF enhancement, but do not allow the independent contribution of each component to be quantitatively resolved. Further stepwise studies are needed to clarify their respective roles and interactions. Biocompatibility is fundamental for the in vivo application of materials. The favorable cytocompatibility and proregenerative effects of F-Fe_3_O_4_ derive not only from the magnetic core but also from Mg^2+^, PDA, and CS. Mg^2+^ improves cell adhesion, stimulates differentiation, accelerates migration, and promotes angiogenic and immunomodulatory processes supporting osteogenesis [85–87]. PDA-modified materials are commonly used in bone repair to enhance cell adhesion and create a more favorable osteogenic microenvironment [88]. CS, a natural amino polysaccharide, is widely used to construct biocompatible coatings for biomedical use [89]. Studies have indicated that weak, moderate, and high-intensity SMF exposure can influence cell proliferation, adhesion, and migration [37, 90, 91]; in our study, SMF amplified the promotive effects of F-Fe_3_O_4_ on hBMSCs, facilitating tissue regeneration (Fig. 3). Notably, magnetite particles may influence cellular behavior even without an external magnetic field [92, 93], consistent with the in vitro performance of P-Fe_3_O_4_. Although less potent than F-Fe_3_O_4_, P-Fe_3_O_4_ still exhibited good cytocompatibility and some capacity to enhance proliferation.

For infectious bone defect repair, the antibacterial performance of biomaterials is crucial. In the F-Fe_3_O_4_ system, both CuO and CS may contribute to the antibacterial activity. However, because their individual effects were not separately evaluated, the observed antibacterial performance should be interpreted as the integrated effect of the multifunctional composite rather than being attributed to any single component. CuO can exhibit broad-spectrum activity through both contact-mediated membrane damage and Cu ion release; the released Cu ion further contributes to bacterial killing by inducing ROS generation via Fenton-like reactions and interfering with bacterial enzymes and DNA, even at relatively low concentrations [94–96]. Previous studies have demonstrated that the size of CuO nanoparticles can influence their antibacterial activity, with smaller nanoparticles generally exhibiting enhanced antibacterial effects due to their higher specific surface area, increased interaction with bacterial membranes, and potentially enhanced Cu ion release [97–99]. Therefore, the CuO (≤ 50 nm) used for F-Fe_3_O_4_ were considered as one of the functional components contributing to the antibacterial and biological effects of the whole system. However, the bulk CuO loading in F-Fe_3_O_4_ should be quantitatively determined in the future. Because copper ions exert concentration-dependent effects, i.e., bactericidal at high levels but potentially cytotoxic when excessive, controlled immobilization and release are critical [100]. The biphasic Cu release pattern, characterized by an early burst followed by sustained release, suggests that F-Fe_3_O_4_ delivers strong initial antibacterial signaling while minimizing later cytotoxic risk [101]. Additionally, the incorporation of CS and CuO could contribute to enhance early-stage antibacterial action while maintaining biocompatibility. Protonated amino groups in the CS shell interact with negatively charged bacterial surfaces, disrupting membrane integrity and permeability [102]. Huong et al. [103] confirmed the feasibility of combined CuO and CS antibacterial strategies. In vitro assays demonstrated antibacterial activity of F-Fe_3_O_4_ against *E. coli*, *S. aureus*, and *E. faecalis* (Figs. 4 and S2), which means that the intrinsic physicochemical and antibacterial properties of the incorporated components of contribute to the antibacterial effect of F-Fe_3_O_4_ [35].The F-Fe_3_O_4_+SMF generally showed lower bacterial survival, although the difference did not reach statistical significance for all three bacterial strains, which demonstrated a trend toward enhanced antibacterial performance under ~ 100 mT SMF. In previous studies, Li et al. [33] investigated SMF-enhanced antibacterial mechanisms with a strength of 250 mT via ROS production, Zhang et al. [30] reported that SMF (500 mT) combined with paramagnetic calcium–polypyrrole nanoparticles significantly increased ROS generation and bactericidal efficacy, and Zhang et al. [34] reported that strong SMF (up to 9 T) induces mutations in *E. coli* by elevating intracellular superoxide radical production. It could be speculated that the contribution of SMF to antibacterial performance in our system was relatively limited may be related to the relatively low magnetic field intensity, underscoring that the strength of SMF in subsequent studies could be increased to explore its correlation with the antibacterial performance. In addition, the physiochemical properties as well as the antibacterial characteristics of the material may change under the influence of SMF. Therefore, SMF may serve as a supportive factor rather than the dominant antibacterial factor in our system. The directly examination of ROS generation, bacterial membrane damage, ion release, and particle–bacterium interactions under SMF conditions in further studies will be valuable for comprehensively understanding the SMF-associated antibacterial mechanisms. Other studies integrating Fe_3_O_4_@PDA or CuO with near-infrared irradiation therapy to enhance antibacterial efficacy provide additional perspectives for optimizing the PDA layer and CuO within the F-Fe_3_O_4_ system [46, 80, 104].

F-Fe_3_O_4_ exhibited favorable in vitro osteogenic effects on hBMSCs (Fig. 5), attributable to the system’s metal and organic components. The widespread application of magnesium-based materials in bone engineering supports the reliability of Mg^2+^. Mg^2+^ release provided sustained, mild ionic stimulation to regulate the osteogenic microenvironment [105]. Fe ions may likewise contribute to persistent microenvironmental or signaling modulation [106]. Low concentrations of copper ions in bone repair materials have also been reported to support osteogenesis, angiogenesis, and immune regulation [101, 107]. Additionally, the positive roles of PDA and CS in bone formation have been confirmed in numerous bone tissue engineering studies [88, 89]. Infectious bone defects are often accompanied by an imbalanced immune microenvironment, which plays a critical role in bone regeneration [13]. Although this study mainly addressed antibacterial and direct osteogenic effects without in-depth immune evaluation, previous studies have shown that the components of F-Fe_3_O_4_ such as Mg and Cu ions possess immunomodulatory properties [108, 109]. Therefore, a systematic evaluation of the immunomodulatory effects of F-Fe_3_O_4_ will be conducted in future research. The present in vitro study showed that F-Fe_3_O_4_+SMF exhibited greater osteogenic capacity than F-Fe_3_O_4_ alone, supporting an additional osteogenic effect of SMF in the context of F-Fe_3_O_4_ treatment [31, 110]. Wu et al. [48] revealed that the addition of 100mT SMF can also improve the involvement of exosomal miRNAs in bone regeneration. Under SMF exposure, magnetic nanoparticles can generate nanoscale magnetic cues that activate osteogenic differentiation and, in some systems, suppress osteoclast activity [27, 111]. However, the current experimental design does not fully distinguish the independent effect of SMF from its interaction with the magnetic particles or the response of F-Fe_3_O_4_ to SMF. Moreover, because SMF-induced changes in the physicochemical properties of F-Fe_3_O_4_ were not directly examined, SMF is primarily considered here as an external physical stimulus acting on the magnetic particle system [112].

In vivo, the F-Fe_3_O_4_+ SMF group demonstrated the most pronounced early antibacterial activity and osteogenic repair-promoting effects, followed by F-Fe_3_O_4_ (Figs. 6 and 7). Previous studies have shown that applying a magnetic field can enhance local particle retention [24], thereby extending therapeutic duration. Injectable GelMA hydrogel was used as a carrier to load the composite particles in the in vivo study, enabling F-Fe_3_O_4_ to continuously exert therapeutic functions. This was due to the widely recognized properties of GelMA, including excellent biocompatibility and photocrosslinkable gelation characteristics [113]. Studies have shown that GelMA hydrogel gradually degrades in the body over 2 to 4 weeks, which matches the time window for bone regeneration, making it suitable as a carrier for various nanomaterials or metal ions [113–115]. In our in vivo experiments, no significant differences were observed between the Blank and GelMA groups in the evaluated outcomes, suggesting that GelMA alone did not exert a detectable independent effect under the present experimental conditions. In contrast, incorporation of F-Fe_3_O_4_ into GelMA significantly improved the therapeutic outcomes, supporting the composite particles as the major source of the observed functional effects. However, the degradation kinetics of GelMA, the release kinetics of F-Fe_3_O_4_ and associated ions from GelMA, and the independent biological contribution of GelMA were not specifically characterized in the present study. In particular, ion release under SMF and non-SMF conditions with or without hydrogel was not directly compared. Previous studies have suggested that the release of metal ions, such as Mg^2+^ and Cu^2+^, is governed primarily by the chemical properties of the coating materials (e.g., CS and PDA) and the surrounding solution environment [116]. From a chemical-equilibrium perspective, therefore, SMF would not be expected to fundamentally alter these underlying dissolution and ion-release processes. However, SMF may indirectly modulate release kinetics. For example, magnetic-field-induced aggregation of magnetic nanoparticles could reduce their effective surface area, whereas magnetic gradient forces may restrict the diffusion of released ions in the vicinity of the particle surface, potentially leading to a modest reduction in the apparent release rate [117]. Although the applied magnetic field here was SMF rather than AMF, which is commonly employed to induce magnetic heating and trigger thermally responsive release, a potential contribution of SMF-induced changes in Fe, Mg, or Cu release cannot be excluded. Future studies on detailed characterization of GelMA’s biological properties, degradation and particle-release behavior with or without SMF will be necessary to better distinguish carrier-related and SMF-related effects. Short-term biosafety assessments indicated robust biocompatibility and promising translational potential for magnetic particles (Figs. S3–5), consistent with the established consensus [19]. Consequently, the copper component in F-Fe_3_O_4_ may minimize the toxicity risks and help regulate a proregenerative microenvironment through low-level release after infection control, thereby facilitating tissue repair during the whole process [107, 118]. It should be noted that, in order to evaluate the early antibacterial response during the acute phase of the infected bone defect model, the 1-week time point was selected for the in vivo study of antibacterial efficacy. Therefore, the present in vivo antibacterial assessment mainly reflects early-stage infection control and cannot guarantee long-term bacterial eradication or exclude persistent low-level infection. Serial bacterial quantification, long-term culture or molecular detection of residual bacteria will be required to clarify the mechanism of SMF-associated enhancement.

Bone formation and homeostasis require substantial energy, with glucose serving as the primary substrate [119–121]. Wei et al. [122] reported that osteoblasts have a high glucose demand; GLUT1 expression precedes RUNX2 expression during osteogenesis, with RUNX2 in turn promoting further GLUT1 expression. Besides, Lee et al. [123] discovered that knocking out *SLC2A1* in mouse chondrocytes severely inhibited cell proliferation and hypertrophy, resulting in significant limb shortening. Together, these studies demonstrate the tight coupling between bone and glucose metabolism. The results of the rescue and pathway blockade experiment in this study indicated that knockdown of *SLC2A1* inhibited the HIF-1 and PI3K/AKT signaling pathways and osteogenic differentiation of hBMSCs. Moreover, inhibition or activation of the HIF-1 and PI3K/AKT pathways also affected the expression of *SLC2A1* and the osteogenic differentiation of cells. Because the bone repair microenvironment is often characterized by hypoxia and nutrient stress, the HIF-1 pathway, a central transcriptional regulator of oxygen homeostasis, can be activated to stabilize HIF-1α and upregulate genes involved in glucose uptake, glycolysis, metabolic adaptation, thereby contributing to a repair phenotype with high bone mass [124–128]. Furthermore, the PI3K/AKT pathway, activated by growth factors and cytokines, generates PIP3 to recruit and activate AKT, thereby regulating nutrient uptake, anabolic metabolism, and cell fate [129, 130]. This pathway is crucial for osteoblast differentiation and skeletal homeostasis. Numerous studies have shown that the PI3K/AKT pathway intersects with HIF signaling, strengthening the integration of metabolic adaptation and signal transduction [131, 132]. Therefore, it can be inferred that in this study, the combined effect of F-Fe_3_O_4_ and SMF may have facilitated *SLC2A1*, the HIF-1 and PI3K/AKT pathways, as well as RUNX2, to form a coordinated regulatory network, driving the energy metabolism remodeling, oxidative stress adaptation, growth, proliferation and osteogenic differentiation of hBMSCs. Notably, Liu et al. [57] reported that 100 mT SMF activates PI3K/AKT signaling and upregulates the key regulatory protein alpha-B crystallin, thereby promoting RUNX2 expression and initiating osteogenic differentiation of hBMSCs, while in endothelial cells, they also found that magnetic scaffolds subjected to SMF stimulation upregulated HIF-1α expression leading to Vascular Endothelial Growth Factor production and angiogenic activation, thereby further enhancing bone regeneration. These findings align with the metabolic and signaling changes observed in the current study under combined F-Fe_3_O_4_ and SMF treatment, supporting a mechanism centered on the *SLC2A1*, HIF-1, and PI3K/AKT axes, which further confirm that the magnetic material system combined with SMF can promote bone regeneration by regulating multiple pathways. The physical stimulation SMF provides could enhance the bidirectional positive feedback among *SLC2A1*, HIF-1, and the PI3K/AKT signaling pathways, amplifying cellular signaling responses and boosting metabolic activity, thereby ultimately accelerating the osteogenic differentiation of hBMSCs. However, similar to the in vitro osteogenic evaluation, the mechanistic experiments did not include an SMF-only group; therefore, although the results support the involvement of *SLC2A1*/HIF-1 and PI3K/AKT signaling in the osteogenic response to F-Fe_3_O_4_+SMF, they do not distinguish the independent contribution of SMF or establish that these signaling changes are SMF-specific or synergy-specific. Prussian blue staining showed iron-positive particles in cells treated with P-Fe_3_O_4_ and F-Fe_3_O_4_, suggesting cellular association/internalization. Importantly, no obvious difference in staining intensity or intracellular distribution was observed after SMF exposure. Although this observation is qualitative, it suggests that the enhanced proliferation, adhesion, migration, and osteogenic differentiation in the F-Fe_3_O_4_+SMF group are unlikely to result simply from increased particle uptake. Based on this observation and previous studies of magnetic biomaterials [133–136], we propose that the additional effect of SMF may involve physical interactions between the magnetic particles and cells, potentially including magneto-mechanical cues at the particle–cell interface and subsequent cellular mechanotransduction. However, these upstream physical processes were not directly examined in the present study. In future work, importantly, the upstream mechanisms linking the material systems, SMF, and pathway activation (such as ion release, ROS generation, mechanotransduction) need to be further investigated.

The main strength of this study lies in integrating antibacterial, osteogenic, biocompatible, and magnetic-responsive functions within a single Fe_3_O_4_-based platform. It also demonstrated that the therapeutic efficacy of multifunctional F-Fe_3_O_4_ particles depends not only on intrinsic material properties but also on the tunable external factor SMF. The application of SMF provides an additional non-contact regulatory stimulus and improves the controllability and parameter optimization of this strategy. Currently, systematic evidence demonstrating the enhancement of antibacterial and osteogenic outcomes via SMF combined with magnetic particles in infectious bone defects remains limited. The present study establishes a relatively coherent evidence chain from material design and bacterial inhibition to cellular responses, providing a conceptual framework for understanding how magnetic particles combined with SMF drive hBMSC osteogenic differentiation. Beyond infectious bone defects, this strategy may have broader potential in infection-associated and magnetically assisted bone regeneration, although further validation and safety evaluation are required.

This study has several limitations that should be addressed in future work. Regarding the material system, the incorporation of PDA increased the tendency of particle aggregation during fabrication, thereby potentially raising cytotoxicity and biosafety concerns. The loading amount and distribution of CuO also warrant improvement. Future studies should optimize modification and preparation processes and explore broader applications by incorporating carriers such as injectable hydrogels or porous scaffolds for sustained-release delivery. Regarding SMF, only a single field strength was evaluated and spatial field uniformity was not systematically characterized. In vivo, because the animals moved freely relative to the magnets, individual magnetic-field exposure was likely variable. In addition, the absence of an SMF-only control prevents clear separation of the intrinsic effects of SMF from the response of F-Fe_3_O_4_ to SMF and their potential interaction. Subsequent research should incorporate systematic magnetic field mapping, confined magnetic exposure configuration, SMF-only controls, and systematical comparisons of different SMF parameters, including intensity and exposure duration to establish clearer parameter–effect relationships and clarify the specific contribution of SMF. Also, the selection of working concentration of F-Fe_3_O_4_ was based on preliminary qualitative morphological screening rather than a systematic quantitative dose–response analysis. Therefore, further concentration-dependent assessment of cell viability and functional responses will be required to more rigorously optimize the working concentration. Although this study mainly assessed antibacterial and osteogenic endpoints, future work could also include intracellular ROS levels, inflammatory factor expression, immune response mechanisms, and macrophage-mediated immune regulation and polarization phenotypes, thereby strengthening the evidence base. Moreover, current animal experiments can only verify short-term preliminary effects, and the histological and osteogenic evidence for bone repair is limited due to specific immune-related markers were not examined, hindering further assessment of the local immune microenvironment and its relationship with bone regeneration. Future studies should incorporate longer-term microbiological monitoring, immunohistochemical or immunofluorescence characterization of local immune responses, and long-term evaluation of material fate and systemic safety. Validation in large animal models will also be necessary to more comprehensively assess therapeutic durability, biosafety, and translational potential.

## Conclusion

The multifunctional magnetic particle system F-Fe_3_O_4_ developed for infectious bone defects achieved two central objectives: effective antibacterial activity and robust osteoinduction. Built upon a magnetic core, the system incorporates SMF as an external modulator to improve controllability and optimization, offering a practical framework for magnetic-based therapies targeting infected bone defects. Under SMF, F-Fe_3_O_4_ not only enhanced bacterial suppression and promoted hBMSC adhesion, migration, and osteogenic differentiation, but also produced favorable therapeutic outcomes in rat femoral infectious defects. Mechanistically, SMF combined with F-Fe_3_O_4_ can regulate *SLC2A1*-mediated glucose uptake and energy metabolism while activating HIF-1 and PI3K/AKT signaling, thereby accelerating the osteogenic differentiation of hBMSC.

## Supplementary Information

Below is the link to the electronic supplementary material.

Supplementary Material 1

## Data Availability

All data supporting the findings of this study are available within the paper and its Supplementary Information.

## Abbreviations

α-MEM: Alpha-minimum essential medium
AKT: Protein Kinase B
ALP: Alkaline phosphatase
AMF: Alternating magnetic field
ARS: Alizarin Red S
BET: Brunauer–Emmett–Teller
BHI: Brain Heart Infusion broth
BV/TV: New bone volume to total tissue volume ratio
CFU: Colony-forming units
CLSM: Confocal laser scanning microscope
CS: Chitosan
CuO: Copper oxide
DEGs: Differentially expressed genes
DLS: Dynamic light scattering
*E. coil*: *Escherichia coli*
*E. faecalis*: *Enterococcus faecalis*
EDS: Energy-dispersive X-ray spectrometer
EG: Ethylene glycol
F-Fe_3_O_4_: P-Fe_3_O_4_-Mg^2+^@PDA-CuO@CS
FBS: Fetal bovine serum
FeCl_3_•6H_2_O: Iron(III) chloride hexahydrate
FT-IR: Fourier transform infrared spectrometer
GelMA: Methacrylate gelatin
GLUT1: Glucose transporter 1
GO: Gene Ontology
GSEA: Gene Set Enrichment Analysis
H&E: Hematoxylin–eosin
hBMSCs: Human bone marrow-derived mesenchymal stem cells
HIF-1α: Hypoxia-inducible factor 1-alpha
ICP: Inductively coupled plasma
KEGG: Kyoto Encyclopedia of Genes and Genomes
LB: Luria–Bertani broth
Mg^2+^: Magnesium ions
MgCl_2_: Magnesium chloride
Mo: Molybdenum
NaAc: Sodium acetate anhydrous
NdFeB: Neodymium-iron-boron
OCN: Osteocalcin
OD: Optical density
OM: Osteogenic medium
PAA: Poly(acrylic acid)
PB: Prussian blue
PDA: Polydopamine
P-Fe_3_O_4_: Poly(acrylic acid)-modified Fe_3_O_4_
PM: Proliferation medium
qRT-PCR: Quantitative reverse transcription polymerase chain reaction
RNA-seq: RNA sequencing
ROS: Reactive oxygen species
RT: Room temperature
RUNX2: Runt-related transcription factor 2
*S. aureus*: *Staphylococcus aureus*
SD: Standard deviation
SEM: Scanning electron microscope
SMF: Static magnetic field
Tb.N: Trabecular bone number
Tb.Sp: Trabecular separation
Tb.Th: Trabecular thickness
TEM: Transmission electron microscope
TGA: Thermogravimetric analyzer
VSM: Vibrating sample magnetometer
XPS: X-ray photoelectron spectrometer
XRD: X-ray diffractometer
