# Supplementary material for: Multifunctional composite magnetic particles combined with static magnetic field for treating infectious bone defects via SLC2A1-mediated HIF-1 and PI3K/AKT pathways
Source: Discov Nano. 2026 Oct 31;21(1):582. doi: 10.1186/s11671-026-04923-w (PMC13723484; doi:10.1186/s11671-026-04923-w)
Supplement: Supplementary file 1 [file 11671_2026_4923_MOESM1_ESM.docx]

**Multifunctional composite magnetic particles combined with static magnetic field for treating infectious bone defects via *SLC2A1*-mediated HIF-1 and PI3K/AKT pathways**

**Mingming Huang ^1,2^, Yuqi Li ^1,2^, Ludan Qin ^2,3^, Xin Gao ^1,2^, Yiyang Tang ^4^, Tian-Yun Huang ^4,5,^*, Jianmin Han ^1,2,^*, Menglong Hu ^6,^***

^1^	Department of Prosthodontics, Peking University School and Hospital of Stomatology & National Center for Stomatology & National Clinical Research Center for Oral Diseases & National Engineering Research Center of Oral Biomaterials and Digital Medical Devices& Beijing Key Laboratory of Digital Stomatology & NHC Key Laboratory of Digital Stomatology & NMPA Key Laboratory for Dental Materials, Beijing, PR China

^2^	Tianjin Medical University School and Hospital of Stomatology & Tianjin Key Laboratory of Oral Soft and Hard Tissues Restoration and Regeneration, Tianjin, PR China

^3^	Department of Prosthodontics, School and Hospital of Stomatology, Cheeloo College of Medicine, Shandong University & Shandong Key Laboratory of Oral Tissue Regeneration & Shandong Engineering Laboratory for Dental Materials and Oral Tissue Regeneration & Shandong Provincial Clinical Research Center for Oral Diseases, Jinan, PR China

^4^	Laboratory for Micromachine Intelligence, School of Advanced Manufacturing and Robotics, PKU Research Center for Robotics, Peking University, Beijing, PR China

^5^	National Key Laboratory of Advanced Micro and Nano Manufacture Technology, Beijing, PR China

^6^	Department of Prosthodontics, Nanjing Stomatological Hospital, Affiliated Hospital of Medical School, Institute of Stomatology, Nanjing University, Nanjing, 210008, PR China

*****	Correspondence: T.-Y.H. (huangtianyun@pku.edu.cn), J.H. (hanjianmin@bjmu.edu.cn), M.H. (dental_hu@bjmu.edu.cn)

Supporting information consists of 8 pages, including this one. There are 5 Figures and 2 Tables.

**Figure S1** Characterization of magnetic particles.

(a) Pore size distribution of Fe_3_O_4_ and P-Fe_3_O_4_ determined by the Density Function Theory method; (b) overlay of XRD patterns of P-Fe_3_O_4_ and F-Fe_3_O_4_; (c) FT-IR spectra of P-Fe_3_O_4_, P-Fe_3_O_4_-Mg^2+^@PDA-CuO, and F-Fe_3_O_4_; (d) XPS survey spectra of P-Fe_3_O_4_, P-Fe_3_O_4_-Mg^2+^@PDA-CuO, and F-Fe_3_O_4_; (e) high-resolution Fe 2p XPS spectrum of P-Fe_3_O_4_-Mg^2+^@PDA-CuO

**Figure S2** *In vitro* antibacterial activity of different magnetic particle groups against *E. faecalis*.

(a) The growth curve of *E. faecalis* within 12 h; (b) images of *E. faecalis* colonies on agar plates; (c) bacterial viability of *E. faecalis*; (d) SEM images of bacterial morphology; (e) CLSM images of live/dead bacterial staining; (f) quantitative analysis of the fluorescence intensity of the live/dead staining. Data throughout are means ± SD (n = 3). **p* < 0.05; ***p* < 0.01; ****p* < 0.001; ns: not significant, *p* > 0.05

**Figure S3** H&E staining of the major organs from Sprague–Dawley rats of different magnetic particle groups at 1 week post-operation

**Figure S4** H&E staining of the major organs from Sprague–Dawley rats of different magnetic particle groups at 4 weeks post-operation

**Figure S5** H&E staining of the major organs from Sprague–Dawley rats of different magnetic particle groups at 8 weeks post-operation

**Table S1** Primer sequences for RT-qPCR

| **Gene** | **5’**–**3’ sequence** | **3’**–**5’ sequence** |
| --- | --- | --- |
| *ALP* | AACATCAGGGACATTGACGTG | GTATCTCGGTTTGAAGCTCTTCC |
| *RUNX2* | CCGCCTCAGTGATTTAGGGC | GGGTCTGTAATCTGACTCTGTCC |
| *BGLAP* | CACTCCTCGCCCTATTGGC | CCCTCCTGCTTGGACACAAAG |
| *SPP1* | GAAGTTTCGCAGACCTGACAT | GTATGCACCATTCAACTCCTCG |
| *SLC2A1* | ATTGGCTCCGGTATCGTCAAC | GCTCAGATAGGACATCCAGGGTA |
| *AKT2* | ACCACAGTCATCGAGAGGACC | GGAGCCACACTTGTAGTCCA |
| *HIF1A*  *GAPDH* | GAACGTCGAAAAGAAAAGTCTCG  CTGGGCTACACTGAGCACC | CCTTATCAAGATGCGAACTCACA  AAGTGGTCGTTGAGGGCAATG |

Abbreviations: *ALP*, Alkaline Phosphatase; *RUNX2*, Runt-related transcription factor 2; *BGLAP*, Bone Gamma-Carboxyglutamate Protein; *SPP1*, Secreted Phosphoprotein 1; *SLC2A1*, Solute Carrier Family 2 Member 1; *AKT2*, AKT Serine/Threonine Kinase 2; *HIF1A*, Hypoxia Inducible Factor 1 Subunit Alpha; GAPDH, Glyceraldehyde-3-Phosphate Dehydrogenase

**Table S2** Summary of the surface area, pore volume, and pore size as determined from N_2_ adsorption–desorption isotherms

|  | **Fe_3_O_4_** | **P-Fe_3_O_4_** |
| --- | --- | --- |
| BET surface area (m²/g) | 28.32 | 40.33 |
| Desorption average pore diameter (4V/A by BET) (nm) | 9.22 | 5.13 |
| Adsorption average pore diameter (4V/A by BET) (nm) | 17.68 | 9.51 |
| t-Plot micropore area (m²/g) | 1.15 | 10.17 |
| t-Plot micropore volume (cm³/g) | 0.00002 | 0.00447 |
| Pore volume at P/Po ≈ 0.99 (cm³/g) | 0.13 | 0.10 |
| Volume in Pores < 1.483 nm (DFT Pore Size) (cm³/g) | 0.00000 | 0.00301 |
